# Context-dependent abstraction of temporal relationships during naturalistic episodic memory

**DOI:** 10.1016/j.isci.2026.117391

**Published:** 2026-09-04

**Authors:** Qun Ye, Yi Hu, Yixuan Ku, Kofi Appiah, Sze Chai Kwok

**Affiliations:** 1 Shanghai Key Laboratory of Brain Functional Genomics, Key Laboratory of Brain Functional Genomics (Ministry of Education), School of Psychology and Cognitive Science, East China Normal University, Shanghai 200062, China; 2 Zhejiang Key Laboratory of Intelligent Education Technology and Application, Zhejiang Philosophy and Social Science Laboratory for the Mental Health and Crisis Intervention of Children and Adolescents, School of Psychology, Zhejiang Normal University, Jinhua 321004, China; 3 Guangdong Provincial Key Laboratory of Adolescent Mental Health and Psychological Development, Center for Brain and Mental Well-Being, Department of Psychology, Sun Yat-sen University, Guangzhou 510006, China; 4 Peng Cheng Laboratory, Shenzhen 518000, China; 5 Department of Computing Science, University of York, YO10 5DD York, UK; 6 Phylo-Cognition Laboratory, Duke Kunshan University - The First People’s Hospital of Kunshan Joint Brain Sciences Laboratory, Division of Natural and Applied Sciences, Digital Innovation Research Center, Duke Kunshan University, Duke Institute for Brain Sciences, Kunshan 215316, China

**Keywords:** mnemonic “pattern-based” code, autobiographical memory, precuneus, temporal memory, fMRI, transcranial magnetic stimulation

## Abstract

How does the human brain represent how far apart in time two remembered moments occurred? Healthy adults played a first-person interactive video game and subsequently judged the temporal order of pairs of event moments during functional MRI. Using representational similarity analysis, we identified a distributed multivoxel activity pattern in the precuneus and surrounding posterior medial cortex whose representational geometry tracked retrieved temporal-distance magnitude across intervals ranging from less than 1 s to approximately 18 min. This representation could not be explained by perceptual similarity, situational change, or task difficulty, and was stronger for events sharing a common episodic context. Applying inhibitory brain stimulation to the precuneus before retrieval attenuated this representational pattern while leaving overall BOLD activity levels unchanged. These findings suggest that the precuneus contributes to organizing temporal relationships among remembered events, representing temporal distance within structured episodic contexts rather than as a simple metric of elapsed time.

## Highlights

• Temporal relationships are abstracted during naturalistic memory retrieval
• Temporal-order behavior reflects temporal distance and episodic context
• Temporal-distance geometry is stronger within shared episodic contexts
• Precuneus rTMS disrupts the geometry of remembered temporal relationships

## Graphical abstract

**Figure:**
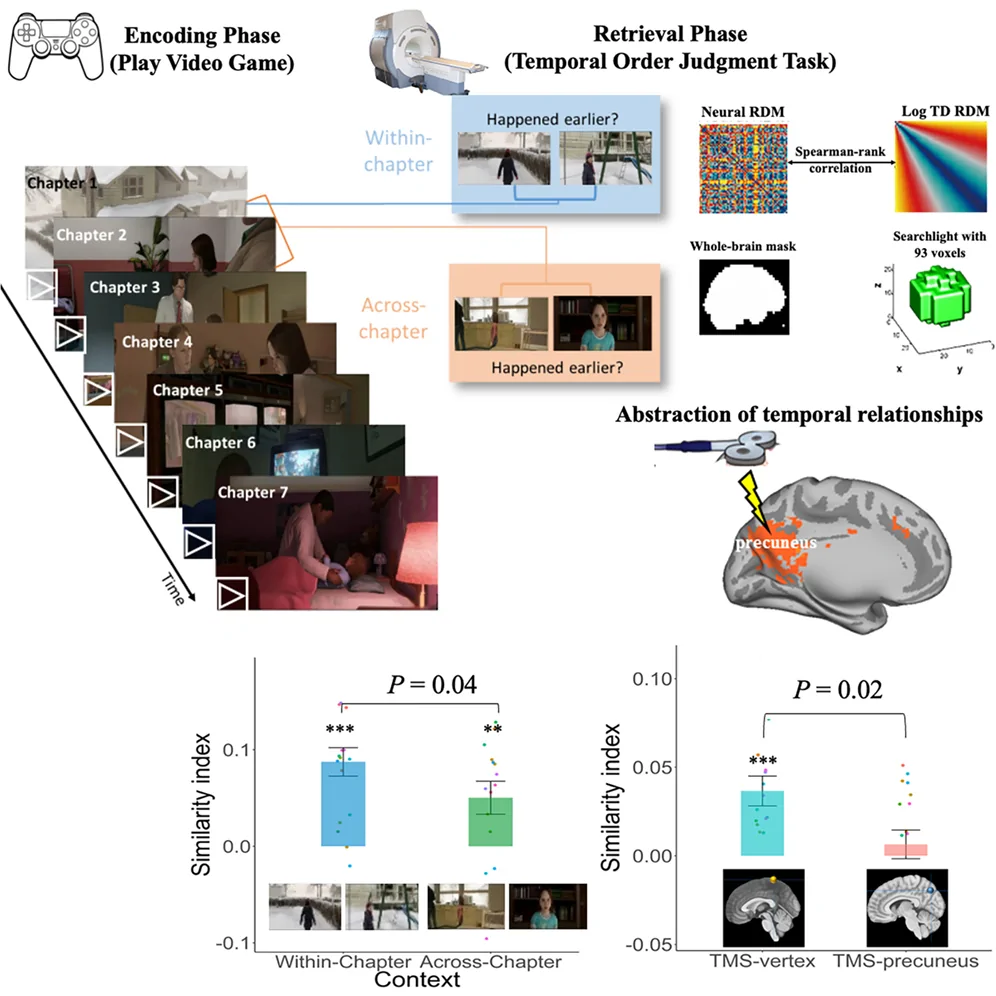

## Teaser

Natural sciences; Biological sciences; Neuroscience; Clinical neuroscience; Cognitive neuroscience

## Introduction

Although physical time can be measured by a clock, the time that the mind reconstructs from memory is not a simple readout of elapsed seconds: experienced and remembered duration can diverge markedly from metric time.^1^^,^^2^ Here we focus on this psychological construct—the temporal distance that separates pairs of remembered events on the seconds-to-minutes scale—and ask how the human neocortex represents it. While the passage of physical time can be biologically registered by distributed sets of brain regions across multiple intervals,^3^^,^^4^ how the human brain abstracts temporal distances separating events in episodic memory remains a significant challenge.^5^

At short timescales, elapsed time can be inferred from single neuron activities in the primate.^6^^,^^7^^,^^8^ Time-registering neurons have been found to code time in the cortico-basal ganglia circuits,^6^ inferior parietal cortex,^7^ and medial temporal lobe.^8^ The overall population state of the lateral entorhinal cortex also encodes temporal information across timescales from seconds to hours.^9^ In contrast, when complex, coherent experiences consolidate into long-term memory,^10^ the neural circuits that build time representations for episodic retrieval are distinct from those implicated in hippocampal-dependent encoding^11^^,^^12^ and retrieval,^13^^,^^14^ and from those supporting transient temporal processing.^6^^,^^7^^,^^8^ While hippocampal ensemble dynamics can form timestamps for temporally close episodes or form distinct timestamps for temporally far episodes in mice,^15^ the pairwise pattern of such representation during retrieval is not completely established at the whole-brain level in humans.

Temporal representation is intertwined with the construction of context.^16^ Item representations are linked to a changing “context” at encoding, such that a common retrieved context is triggered during recall for items that were experienced within a temporal context.^17^ More recent frameworks show that the brain segments continuous experience into discrete events represented by neural population states.^18^ These states form neural “event trajectories”, where the dissimilarity between the neural patterns associated with different events serves as a code for the passage of time^19^ and spatiotemporal context.^14^ Event nodes^20^ and event boundaries^21^^,^^22^^,^^23^ could shape brain activity during encoding. However, contextual effects^24^^,^^25^ on neural activity during retrieval—especially their interplay with temporal separation—have been less reported.

Neural pattern dissimilarity in the prefrontal cortex and hippocampus increases with the temporal separation between items^26^ and between autobiographical events experienced temporally apart.^14^ The growing dissimilarity with longer temporal separation correlates with estimates of elapsed time.^27^^,^^28^ For the recollection of autobiographical memories or episodic events, the posterior medial (PM) memory system, including the hippocampus, precuneus, and angular gyrus, plays an instrumental role.^29^^,^^30^^,^^31^^,^^32^ However, little is known about how the PM system contributes to representing the elapsed time between episodic events.

Temporal-context and drift accounts predict that neural patterns grow increasingly dissimilar with greater temporal separation, yielding a positive, monotonic temporal-distance code.^17^ By contrast, differentiation/repulsion accounts predict the opposite for proximal or overlapping events, whose representations can be actively pushed apart to become less similar than those of distant events, reversing the objective similarity structure.^33^^,^^34^^,^^35^^,^^36^ Whether a graded distance code or a repulsion signature dominates during retrieval—and in which regions—is therefore an open empirical question that our parametric, second-order design is positioned to adjudicate.

Moreover, event boundaries are known to influence episodic encoding and retrieval,^22^^,^^37^^,^^38^ but it remains unclear whether they also shape the neural representation of temporal relationships themselves. Specifically, it is unknown whether temporal distance is represented as a context-independent metric or whether its neural representational geometry is embedded within coherent episodic contexts. These alternatives make different predictions for representational similarity analyses. If temporal distance is encoded as an abstract global metric, representational similarity should be preserved across contextual boundaries. Conversely, if temporal representations are embedded within event structure, temporal distance coding should be selectively enhanced for events belonging to the same episodic context.

**Figure 1:**
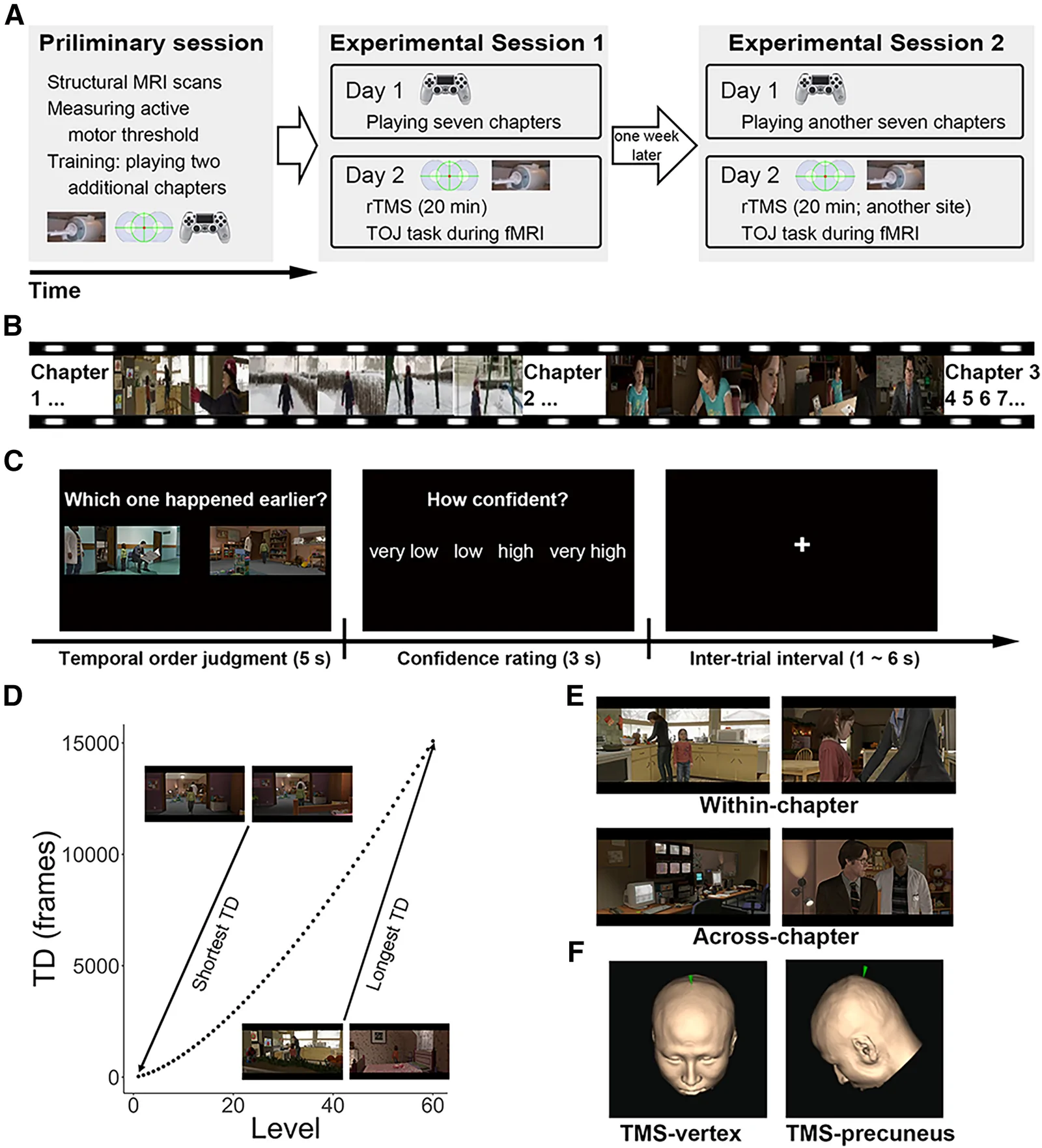
An interactive video-game paradigm for probing temporal-distance representation with concurrent fMRI and rTMS (A) In experimental sessions 1 and 2, participants played a video game containing seven related chapters with a first-person perspective for encoding, and 24 h later, received 20 min of repetitive transcranial magnetic stimulation (rTMS) to either one of two cortical sites before performing a temporal order judgment task during fMRI. Order of TMS sites (within-subjects) and choices of video game chapters were counterbalanced across subjects (Table S1). The two experimental sessions were conducted on different days to minimize rTMS carry-over effects (mean separation = 8 days). Participants underwent structural MRI scans, familiarized themselves with the gameplay using a console, and had their active motor threshold measured (for TMS) on a different day before the experimental sessions proper. (B) Gameplay video: each encoding session consisted of seven chapters (Figure S1). (C) Temporal order judgment task. Participants chose the image that happened earlier in the video game and reported their confidence level. (D) 60 levels of temporal distances (TD) were generated for each subject according to their subject-specific video-playing duration. Although the absolute TDs were different across subjects (Figure S1), we ensured they were scale-invariant using a power function during image selection. Actual TDs from one subject (subj01) are shown. (E) Two pairs of images were extracted from the same chapter (Within-chapter) or two adjacent chapters (Across-chapter). The 60 levels of TD were fully matched within-subjects for these two conditions. Note that scenes depicted in Within-chapter tended to be more contextually similar than those depicted in Across-chapter. (F) TMS stimulation sites, superimposed onto one subject’s MRI-reconstructed skull, are marked by a green pointer. The MNI coordinates for precuneus stimulation: x, y, z = 6, −70, 44.

Thus, our hypothesis is that upon retrieval, pairwise temporal distances between events can be revealed in the form of a multivoxel pattern and that this multivoxel pattern is modulated by contextual coding as enacted at encoding. We examined the abstraction of temporal memories and determined how different regions of this system are implicated in the representation of temporal distances as well as their interaction with episodic contexts. We asked whether temporal relationships are represented as a context-independent metric or are embedded within an episodic structure. Combining naturalistic memory with a first-person interactive video game, RSA, and TMS, we show that temporal representations are context-dependent and causally supported by the posterior medial memory system (Figure 1A).

## Results

### Behavioral results

Overall, participants showed greater accuracy for longer temporal distances (TDs), with this benefit being more pronounced when image pairs originated from the same chapter (Within-chapter) compared to different chapters (Across-chapter). After collapsing across temporal distance (TD) conditions, in the TMS-vertex session, mean accuracy was numerically higher for the Across-chapter condition (*M* = 0.71, *SD* = 0.46) than for the Within-chapter condition (*M* = 0.66, *SD* = 0.47). However, in the TMS-precuneus session, mean accuracy was numerically higher for the Within-chapter condition (*M* = 0.69, *SD* = 0.46) than for the Across-chapter condition (*M* = 0.65, *SD* = 0.48). Regarding mean RTs, in the TMS-vertex session, mean RTs were numerically higher in the Within-chapter condition (*M* = 2.63, *SD* = 0.86) than in the Across-chapter condition (*M* = 2.51, *SD* = 0.87). In the TMS-precuneus session, mean RTs were likewise higher for Within-chapter (*M* = 2.74, *SD* = 0.82) than for the Across-chapter condition (*M* = 2.70, *SD* = 0.87).

**Figure 2:**
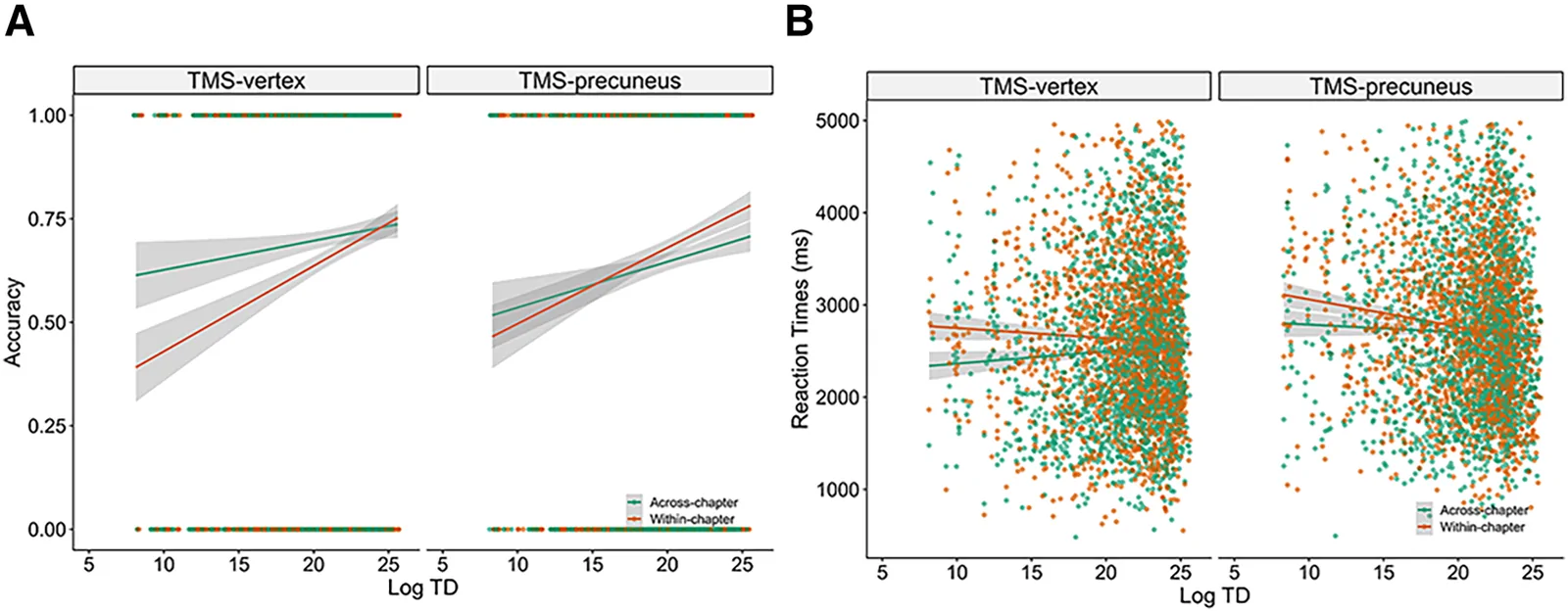
Temporal-order accuracy and reaction timescale with temporal distance and are modulated by episodic context and precuneus stimulation (A) Effects of temporal distance (TD), Context (Across-chapter vs. Within-chapter), and TMS session (TMS-vertex vs. TMS-precuneus) on TOJ accuracy. Both Log TD (in frame) and its interaction with Context significantly predicted accuracy. Accuracy increased with greater Log TD for both Context conditions. However, Within-chapter accuracy (orange line) showed a steeper slope compared to Across-chapter accuracy (green line). The Context by TMS session interaction revealed contrasting patterns: in the TMS-vertex condition, accuracy was higher for Across-chapter (green line), while in the TMS-precuneus condition, accuracy was higher for Within-chapter (orange line). (B) Effects of temporal distance (Log TD), Context (Across-chapter vs. Within-chapter), and TMS session (TMS-vertex vs. TMS-precuneus) on TOJ response times (RTs). Log TD and its interaction with Context significantly predicted RTs. In the TMS-vertex condition, Across-chapter RTs (green line) increased with greater Log TD, while within-chapter RTs (orange line) decreased with Log TD. The Log TD and Context interaction is smaller in the TMS-precuneus condition. Points depict trial-level observations for each participant: in (A), accuracy is coded per trial (1 = correct, 0 = incorrect); in (B), each point is the RT from a single trial. Points are semi-transparent, with a slight horizontal jitter to reduce overlap. The x axis displays log-transformed temporal distance; because spacing is compressed at larger values on a logarithmic scale, dots appear more numerous and concentrated in the long-TD range. Colors indicate Context (Across-chapter = green; Within-chapter = orange). Images are faceted by TMS session (left: TMS-vertex; right: TMS-precuneus). Log TD = log-transformed temporal distance.

A mixed-effects logistic regression examined the effects of TD, Context, and TMS site on accuracy (Figure 2A). The model’s fixed effects explained 1.9% of the variance in accuracy (Marginal *R*^*2*^ = 0.019), while the full model including the random effect explained 4.3% of the variance (conditional *R*^*2*^ = 0.043). TD significantly predicted accuracy, *χ*^*2*^_(1)_ = 86.87, *p* < 0.001, with longer temporal distances associated with higher accuracy (odds ratio = 1.05). Context (*χ*^*2*^_(1)_ = 0.03, *p* = 0.872) and TMS site (*χ*^*2*^_(1)_ = 0.02, *p* = 0.880) showed no significant main effects. A significant TD × Context interaction emerged, *χ*^*2*^_(1)_ = 11.11, *p* < 0.001, indicating that the increased accuracy for longer TDs was steeper for Within-chapter pairs (slope = 0.09, *SE* = 0.01) than for Across-chapter pairs (slope = 0.04, *SE* = 0.01; difference: *z* = 3.33, *p* < 0.001). A significant Context × TMS site interaction was observed, *χ*^*2*^_(1)_ = 17.29, *p* < 0.001. Follow-up analyses revealed contrasting patterns: precuneus stimulation resulted in lower accuracy for Across-chapter conditions compared to Within-chapter conditions (odds ratio = 0.82, *z* = −2.82, *p* = 0.005), whereas there was higher accuracy for Across-chapter compared to Within-chapter conditions with vertex stimulation (odds ratio = 1.24, *z* = 3.06, *p* = 0.002). No significant interaction was found between TD and TMS site (*χ*^*2*^_(1)_ = 0.01, *p* = 0.917), nor was there a significant three-way interaction (*χ*^*2*^_(1)_ = 0.47, *p* = 0.491).

Regarding RTs, a linear mixed-effects model examined the effects of temporal distance (TD), Context, and TMS site on RTs (Figure 2B). TD, Context, and TMS site showed significant main effects: TD, *F*_(1, 7794)_ = 4.43, *p* = 0.035, *η*^*2*^_*p*_ = 0.001; Context, *F*_(1, 7794)_ = 21.61, *p* < 0.001, *η*^*2*^_*p*_ = 0.003; and TMS site, *F*_(1, 7794)_ = 75.71, *p* < 0.001, *η*^*2*^_*p*_ = 0.01. Significant two-way interactions were observed between TD and Context, *F*_(1, 7794)_ = 20.48, *p* < 0.001, *η*^*2*^_*p*_ = 0.003; TD and TMS site, *F*_(1, 7794)_ = 7.83, *p* = 0.005, *η*^*2*^_*p*_ = 0.001; and Context and TMS site, *F*_(1, 7794)_ = 6.68, *p* = 0.01, *η*^*2*^_*p*_ = 0.001. Follow-up analyses revealed that the TD effect on reaction times was significantly more negative for Within-chapter (slope = −0.02, SE = 0.004, *z* = −4.70, *p* < 0.001) than Across-chapter contexts (slope = 0.01, SE = 0.004, *z* = 1.71, *p* = 0.09; difference: *z* = −4.53, *p* < 0.001), indicating RTs decrease with longer TDs within chapters but stay flat across chapters. The TD effect was more negative following precuneus stimulation (slope = −0.01, SE = 0.004, *z* = −3.47, *p* < 0.001) compared to vertex stimulation (slope = 0.002, SE = 0.004, *z* = 0.52, *p* = 0.60; difference: *z* = −2.80, *p* = 0.005). The three-way interaction was not significant, *F*_(1, 7794)_ = 0.23, *p* = 0.631.

These behavioral patterns are replicated with analyses restricted to high-confidence trials only (Figures S2A and S2B).

#### Control analysis on removing effects from long TDs

Although we matched the 60 TD levels within-subjects across Contexts, longer within-chapter TDs were more likely to be extracted from the longest/longer chapters (e.g., Chapter 1, 9, and 12; see Figure S1), raising potential chapter-identity confounds, which could lead to other confounds (e.g., cumulative exposure, primacy). To address this, we performed a control analysis. We re-binned all TD levels to ensure that the “long TDs” would not be prevalently extracted from exceedingly long Chapters (e.g., Chapter 1). Prior to inferential statistical analysis, we excluded trials from the longest temporal distance bin to minimize potential chapter confounds. This binning variable was constructed by dividing each participant’s temporal distance (TD) distribution into six quantiles within each context, with analyses conducted only on the five lower quantiles (TD01 ∼ TD50; TD51-60 discarded). This control analysis did not alter the pattern of behavioral results observed in the original analysis of the full dataset. This analysis revealed significant interactions for Context × TMS, *χ*^*2*^(1) = 17.63, *p* < 0.001, and for TD × Context, *χ*^*2*^(1) = 4.83, *p* = 0.028. We also observed a significant main effect of TD, *χ*^*2*^(1) = 57.55, *p* < 0.001.

### Functional MRI results

#### Representation of temporal distances

First and foremost, we searched for neural representations that resemble the matrix of temporal distances using searchlight representational similarity analysis (RSA).^39^

**Figure 3:**
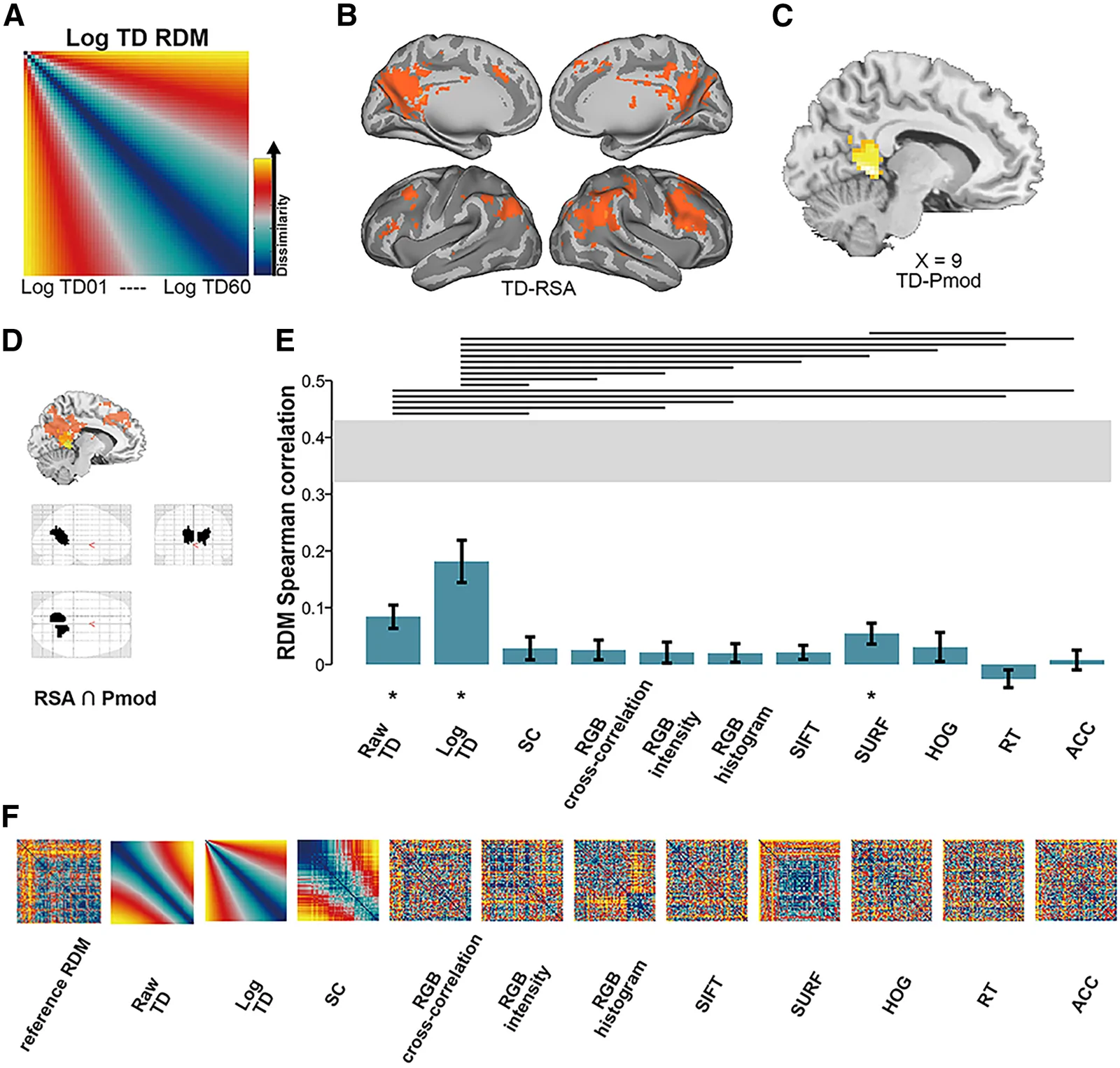
A multivoxel representation of retrieved temporal-distance magnitude in the posteromedial parietal cortex (A) Log TD representational dissimilarity matrix (RDM) for searchlight RSA. The RDM consisted of 60 subject-specific Log TD levels. Any two event-moments that are separated by short TD will become increasingly dissimilar from the other two event-moments as the TD increases. (B) Using Log TD RDM for searchlight RSA, clusters of voxels that contained TD information were primarily in the posteromedial cortex, bilateral angular gyri, and bilateral middle frontal gyri. (C) Activation signal intensity from parametric modulation analysis (Pmod). The intensity of these voxels, primarily in the left precuneus, increased as a function of Log TD. (D) Conjunction region for neural signal extraction for subsequent inferential tests. The mask was created by intersecting the similarity map from RSA and the activation map from Pmod. (E) Inferential comparisons of multiple model representations. Several candidate RDMs were tested and compared for their ability to explain the neural reference RDM extracted from the conjunction mask. Log TD RDM was the best model among these candidate RDMs to explain the variances in the neural reference RDM. Horizontal lines over two bars indicate that the two models have significant statistical differences (signed-rank test) after multiple testing corrections. The gray horizontal bar indicates the expected performance of an unknown true model, given the noise in the data. Asterisks indicate a significant Spearman correlation between the subject-specific reference RDM and candidate RDMs. Error bars indicate the SEM over participants. Asterisks (∗) denote *p* < 0.05, FDR-corrected. (F) Reference (neural) RDM and candidate RDMs. The reference RDM was estimated using the BOLD responses elicited by the stimuli during fMRI in accordance with the 60 TD levels in the conjunction region (only Within-chapter condition displayed). The group-level neural reference RDM shown here is for display purposes (see Figure S4 for individual neural RDMs). Situational Change (SC) RDM was computed by the number of locations each participant had virtually traversed in the video (Figure S1D). Perceptual RDMs (RGB cross-correlation, RGB intensity, RGB histogram, SIFT, SURF, HOG) were computed based on image properties (see details in STAR Methods). Behavioral RDMs (RT and ACC) were computed based on the behavioral performance of reaction times and percentage correct for each trial. MRI results are displayed at *P*_uncorrected_ < 0.001.

For the fMRI results, we first focus on the TMS vertex condition only and then examine the effect of TMS afterward. Without *a priori* bias for any region of interest, we searched across the entire cortex using an RDM consisting of 60 levels of logarithmically transformed subject-specific TD (Figure 3A) and identified clusters of voxels that contain information about the set of geometrically defined temporal distances in memory (see STAR Methods and Figure S3). In our study, importantly, the RSA does not attempt to decode temporal distance directly from neural activity. Rather, it asks whether the geometry of neural population responses preserves the geometry predicted by temporal distance. Our analysis concerns a second-order representational structure: retrieval trials involving similar temporal-distance magnitudes are predicted to evoke similar multivoxel activity patterns, whereas trials involving increasingly different temporal distances are predicted to evoke increasingly dissimilar neural patterns. This second-order representational geometry is the quantity that RSA is specifically designed to evaluate.^40^ Within this 60 × 60 RDM of temporal-distances, we revealed that the neural pattern of judging the temporal order of a pair of memories separated with a given temporal distance is more similar to other temporal order judgements which enclosed temporal distances of a comparable magnitude (adjacent ones, e.g., TD1 more similar to TD2 than to TD60). These voxels were in the posteromedial parietal areas, bilateral angular gyri, and middle frontal gyri (Figure 3B and Table 1).

**Table 1:** Brain representation associated with Log TD RDM in TMS-vertex session (Figure 3B)

Cluster		Peak			T value	Brain regions
k	*p*-FWE	x	y	z		
7554	<0.001	30	21	38	5.78	right middle frontal gyrus
		30	21	44	5.59	right precuneus
		6	−42	20	5.54	right middle frontal gyrus
		−3	−45	20	5.43	left posterior cingulate cortex
		−6	−42	17	5.39	left posterior cingulate cortex
		60	−48	23	5.14	right supramarginal gyrus
		48	−48	35	5.05	right angular gyrus
		−9	−72	23	5.00	left precuneus
		60	24	14	4.99	right inferior frontal gyrus (pars triangularis)
		−39	−66	35	4.99	left angular gyrus
		−12	−48	11	4.84	left precuneus
		−51	33	17	4.81	left inferior frontal gyrus (pars triangularis)
		51	9	32	4.80	right precentral gyrus

Having identified a multivariate pattern underlying the temporal memory abstraction in the posterior medial parietal cortex, we asked whether there were voxels whose activities change monotonically as a function of temporal distance using a standard univariate approach, irrespective of their multivoxel characteristics. A whole-brain parametric modulation analysis revealed log TD-specific BOLD signals in a cluster within the posteromedial region, including the precuneus (Figure 3C and Table 2). Importantly, since the two types of analyses extracted two different kinds of neural information, their overlap in the precuneus jointly confirms the critical involvement of this region. We accordingly created a conjunction map (Figure 3D), so that both the multivariate and univariate results underlying the temporal distance abstraction would be available for the next analysis.

**Table 2:** Brain activation parametrically modulated by Log temporal distance (TD) in TMS-vertex session (Figure 3C)

Regressor	Cluster		Voxel				Brain Regions
	k	*p*-FWE	Z value	x	y	z	
TD	555	<0.001	4.62	9	−45	−1	right cerebellum (lobules IV–V)
			4.35	−6	−57	20	left precuneus
			4.27	6	−54	5	cerebellar vermis (lobules IV–V)
	116	0.002	3.87	36	−81	14	right middle occipital gyrus
			3.78	42	−72	29	right middle occipital gyrus
			3.75	36	−69	11	right middle occipital gyrus
	117^a^	0.01	4.34	45	−36	5	right superior temporal gyrus
			4.17	54	−36	5	right superior temporal gyrus
			3.81	48	−27	−1	right superior temporal gyrus

a Denotes a negative relationship between TD and brain activity.

We then performed inference tests to statistically assess whether the Log TD RDM is significantly correlated with subject-specific neural RDM (labeled as reference RDM in Figure 3F; see also individual neural RDMs per participant in Figure S4) estimated from the fMRI response pattern in accordance with the 60 Log TD levels (4 repetitions per TD level) within this conjunction region. This would allow us to test whether the Log TD RDM could explain the neural representation better than the other candidate RDMs. The Log TD RDM (our best-performing model) accounted for the variance far better than the other candidate RDMs, such as Situational Change (SC) RDM, six perceptual RDMs, and two behavioral RDMs (Figures 3E and 3F). However, the comparison between the Log TD and Raw TD models yielded *p* = 0.975 (FDR-corrected) (rdifference = 0.098), indicating that the two models explained the neural data equally well in the present dataset. Accordingly, the present findings should be interpreted as evidence for a monotonic temporal-distance representation, whereas the precise scaling function (e.g., logarithmic versus raw temporal distance) remains to be determined by future work.

For all subsequent analyses, we retained the Log TD model for the primary analyses because it reflects our *a priori* theoretical hypothesis, which was specified before analysis and motivated both the experimental design and previous work suggesting that subjective temporal distance is represented in a compressed manner rather than linearly.^2^ The Log TD model predicts that neural patterns for temporally close events should be more similar (lower dissimilarity) than patterns for temporally distant events. Our analyses show that Log TD significantly predicts the neural RDM, survives all perceptual and difficulty controls, and approaches the noise ceiling.

#### Control analyses to rule out effects of perceptual similarity and situational shifts

The temporal-distance memory representation could be confounded by perceptual similarity in each pair of images. To address this concern, we conducted six separate RSAs, in which we indexed perceptual similarity between the image-pairs by six different metrics, some drawn from the visual categorization models referred by Greene et al.^41^ and Aminoff et al.^42^ The six RDMs are Red Green and Blue (RGB) cross-correlation RDM, RGB-intensity RDM, RGB-histogram RDM, scale invariant feature transform (SIFT) RDM,^43^ speeded up robust features (SURF) RDM,^44^ and histogram of oriented gradients (HOG) RDM^45^ (see STAR Methods; supplemental information). No similar representation was observed in the posteromedial parietal cortex using these candidate RDMs except the SURF RDM (Figures S5B–S5G). Importantly, to further exclude the contribution of these additional perceptual properties, we conducted a whole-brain searchlight correlation between the Log TD RDM and the brain response pattern, this time regressing out the six perceptual RDMs using a partial Spearman’s correlation analysis method. This partial correlation analysis showed that the effects of abstraction of TD distributed in the posteromedial parietal areas remained even after removing the influences of all these perceptual properties, suggesting that TD multivoxel representation could not be driven simply by perceptual properties (Figure S5J).

Considering previous work on space-time relationships in episodic memory,^14^^,^^46^ we also quantified the space displacement embedded between the image-pairs by computing the number of locations each participant had virtually traversed in the video game and entered them into a subject-specific Situational Changes RDM (Figure S1D). Despite the space-time correlation in the encoding material (Figures S2C and S2D), subject-specific Situational Changes RDMs explained very little variance in comparison to our temporal-distance RDM (Figure S5A). Longer TDs tend to coincide with greater visual dissimilarity and more situational changes. This covariance is expected, given that such features are known cues on which the brain leverages when reconstructing temporal distance.^11^^,^^27^ The key question, therefore, is whether precuneus activity reflects a genuinely integrated temporal-distance signal rather than a simple aggregation of perceptual or situational properties. Our model-comparison and partial-correlation analyses (Figures 3E and S5J) show that it does: the precuneus encodes temporal-distance information that cannot be explained away by these component features. We also confirmed that participants’ behavioral performance RDMs, namely reaction times and accuracy, could not explain the putative temporal-distance representation (Figures S5H–S5I).

#### Robustness of the temporal distance RDM effects

To ensure that the observed representational structure was not driven by group-averaging artifacts or by extreme long-vs-short TD comparisons, we conducted several additional robustness analyses. First, we emphasize that all inferential statistics were performed at the individual-subject level: for each participant, the neural RDM extracted from the conjunction mask was correlated with the candidate model RDMs, and these subject-level correlation coefficients were then entered into group-level statistical tests. Thus, the reported effects do not depend on the group-averaged RDM visualization shown in Figure 3F.

Second, to control any disproportionate influence from long-TD pairs and to rule out any artifact effects driven by the selected part of the RDM (e.g., the shortest TDs) or by extreme long-vs-short comparisons, we repeated the analysis but now iteratively reduced the RDM matrix size using two truncation strategies (see Supplementary Materials^47^ for this approach). We conducted this analysis comprehensively by truncating either the beginning or the end of the RDM. Iteratively removing the shortest or longest temporal-distance bins did not abolish the Log TD-neural relationship, confirming that the effect does not depend on edge bins or on specific regions of the TD spectrum. This truncation analysis confirmed that this “Log TD – Neural” relationship was not an artifact of extreme values at the edges of the TD spectrum. The correlation remained significant even when the shortest or longest TDs were progressively removed, confirming a genuine gradient-like representation (Figure S6). For the Trim Tail strategy, correlations remained statistically indistinguishable from the full-matrix baseline (all *t*(16) < 1.65, *ps* > 0.10, FDR-corrected), even when long TDs were removed down to a matrix size of 18. In contrast, for the Trim Head strategy, correlations began to decline significantly at matrix size = 56 (*t*(16) = 2.77, *p* = 0.007, FDR-corrected). A linear mixed-effects model with trimming strategy and matrix size as fixed effects revealed a significant interaction (*F*(1, 848) = 5.0, *p* < 0.001): the slope of decline was steeper for Trim Head (*β* = 0.0027) than Trim Tail (*β* = 0.0015), confirming that the drop observed for short-TD trimming is statistically reliable. The trimming results do not indicate that the effect is driven only by the shortest TDs; rather, they (1) address the concern that long-TD bins might drive the effect and (2) show that the TD representation is strongest in the short-to-intermediate range, consistent with established psychophysical accounts of compressed temporal coding. These results showed that the neural signals coded in the posteromedial region, as revealed by these searchlight analyses, were most attributable to the mnemonic representation of temporal distances. Together, these analyses demonstrate that the temporal-distance representational structure in the precuneus is robust at the individual-subject level and is not an artifact of group averaging or extreme dissimilarity values (Text S1).

#### TD representation is context-dependent

**Figure 4:**
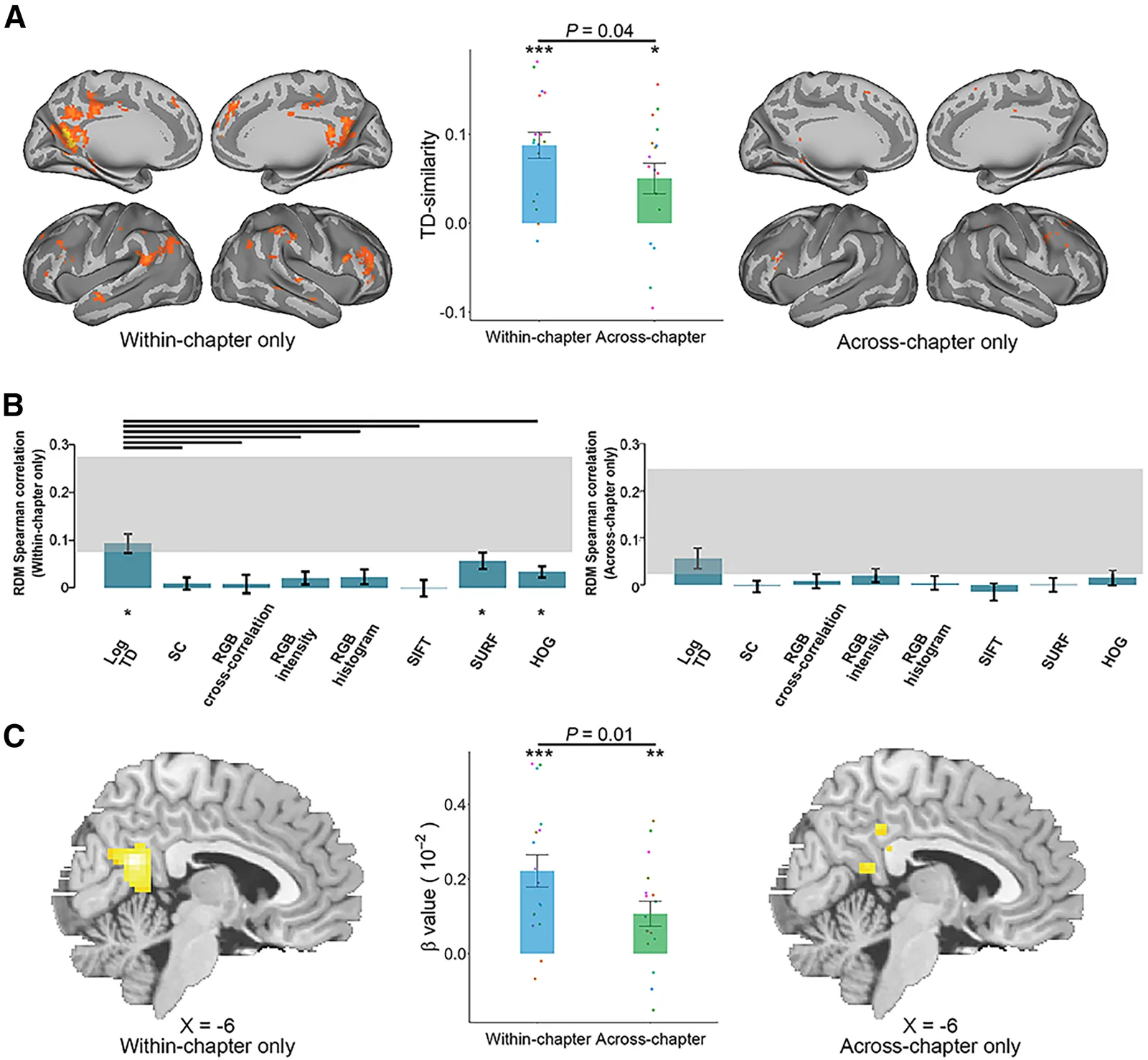
Temporal-distance representations are stronger for events that share an episodic context (A) Stronger multivoxel similarity representing Log TD variation in the Within-chapter condition than in the Across-chapter condition in the conjunction region (middle image). (B) Separate model comparisons show the Log TD RDM is the best model to explain neural variances in the Within-chapter condition (although SURF RDM and HOG RDM are also related to the neural reference RDM), whereas all the candidate RDMs could not explain the neural variances in the Across-chapter condition. (C) Stronger parametric activation intensity in the Within-chapter condition than in the Across-chapter condition. In (A) and (C), each same-color dot represents one subject. Error bars denote the SEM over subjects. ∗∗∗, ∗∗, ∗ denote *p* < 0.001, *p* < 0.01, and *p* < 0.05, respectively, FDR-corrected, one-sample *t* test against zero.

To test the hypothesis that the TD-neural pattern similarity index is higher when the two images are extracted from a shared context than when they are from two different contexts, we ran a new searchlight RSA, separately for the Within-chapter and Across-chapter trials. The representation of TD was observed only in the Within-chapter condition (Figure 4A, left) but not in the Across-chapter condition (Figure 4A, right). The voxels identified by the searchlight RSA were in the precuneus, retrosplenial cortex, and angular gyri bilaterally.

For statistical inference, we extracted the similarity index within the conjunction mask (RSA and pmod maps), using a bias-free leave-one-subject-out method (STAR Methods). In line with our prediction, the voxels in the Within-chapter trials contained higher pattern similarity to the Log TD RDM than Across-chapter trials (Figure 4A, middle image; one-tailed: *p* = 0.04), confirming that the neural pattern similarity related to the Log TD RDM was indeed stronger in Within-chapter trials. We then performed statistical inference tests to assess whether the Log TD RDM explains the neural reference RDM better than other candidate RDMs separately for Within-chapter and Across-chapter conditions (Figure 4B). The Log TD model accounted for the variances far better than the other candidate RDMs in the Within-chapter condition (Figure 4B, left). By contrast, the Log TD RDM (and all other candidate RDMs) failed to explain the neural reference RDM in the Across-chapter condition (Figure 4B, right).

This context-dependent difference was also found in a voxel-wise univariate analysis. The beta-estimates (*β*) from a pmod analysis using Log TD as a regressor were significantly higher in the Within-chapter condition compared to the Across-chapter condition (Figure 4C). These findings indicate that temporal distance representations are selectively expressed when events are retrieved within a common episodic context, suggesting that contextual organization influences the neural coding of temporal relationships. This confirms that the mnemonic representation of temporal distances was determined by whether the pairs of images were experienced within a shared context, corroborating the interaction between temporally and semantically defined factors observed during memory encoding^11^ and retrieval.^13^

#### TMS reduced the precuneal representation of TD

**Figure 5:**
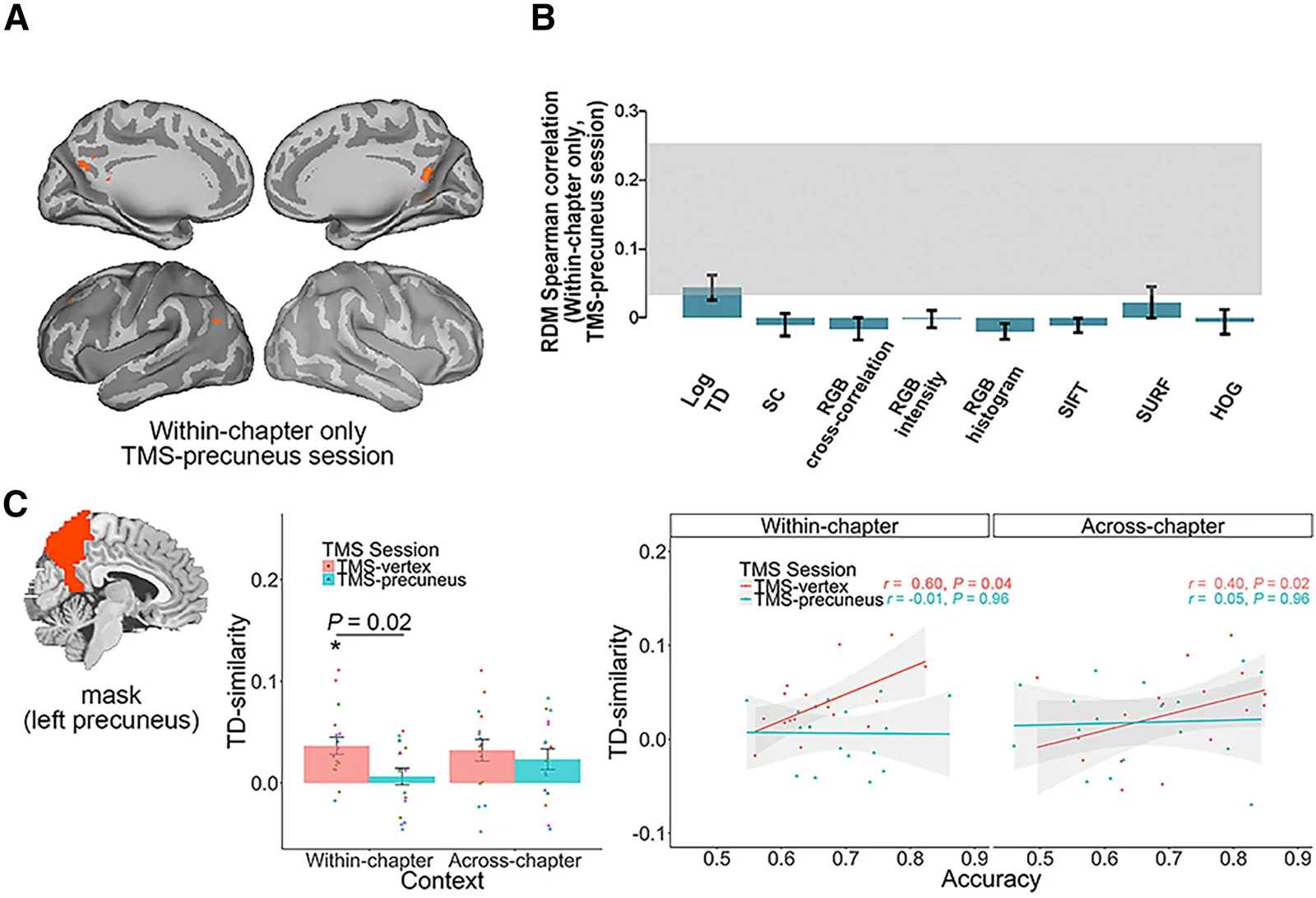
Precuneus rTMS attenuates the multivoxel representation of temporal distance TMS reduced the precuneal representation of TD. (A) Very few voxels survived in the searchlight RSA after TMS on the precuneus (voxel level: *P*_uncorrected_ < 0.001; cluster level: *P*_FWE_ < 0.05). (B) Model comparisons showed that the candidate RDMs explain little neural variance after TMS on the precuneus. (C) Left and middle images: We applied different anatomical masks to extract TD-similarity values from subjects (Figure S7). Among all masks, we found that TMS-precuneus, compared to the TMS-vertex, reduced the temporal distance representation in the precuneus in both hemispheres (left image; see results for the right precuneus in Figure S7A). Right: Taking the left precuneus as an example, correlation analyses showed that TMS on the precuneus weakened the significant neural-behavioral correlation (TMS-vertex session: *r* = 0.60, *p* = 0.04; TMS-precuneus session: *r* = −0.01, *p* = 0.96; comparison between two correlations: *z* = 2.07, *p* = 0.04). *p* values are FDR-corrected. ∗ denotes *p* < 0.05, FDR-corrected, one-sample *t* test against zero.

To ascertain a pivotal role of the precuneus in this memory operation, we deployed a disruptive technique, strategically targeting the precuneus with repetitive transcranial magnetic stimulation to interrogate changes on both neural and behavioral levels (within-subjects: TMS-precuneus vs. TMS-vertex; Figure 1F and Table S1). Precuneus stimulation attenuated the detectable multivoxel representation of retrieved temporal-distance magnitude within the posterior medial region, either considering the Within-chapter condition only (Figure 5A) or collapsing Within-chapter and Across-chapter conditions. Model comparison results showed that the correlation values between all the candidate RDMs and the neural reference RDM now failed to reach statistical significance even when only considering Within-chapter trials alone (Figure 5B).

Inspired by the fractionation view for the lateral parietal cortex^48^ and previous work on the parcellation of the PM memory network,^49^ we tested the possibility that there might be differences in the patterns of neural activity associated with the abstraction of temporal relationships within an episodic context in the sub-regions of the putative PM memory network.^32^ Based on our main MRI results (Figure 3B), we have chosen six anatomical regions-of-interest in the PM memory network (ROIs: bilateral precuneus, bilateral angular gyrus, and bilateral hippocampi; see STAR Methods), together with the primary visual region (entire occipital cortex) as a control, to more finely characterize the disruptive effect caused by the TMS.

We extracted pattern similarity indices from these ROIs and found that the neural-TD pattern similarity in the precuneus in both hemispheres was significantly weakened following TMS to the precuneus specifically for the Within-chapter condition (left precuneus: Figure 5C left and middle images, one-tailed: *p* = 0.02; right precuneus: Figure S7A, one-tailed: *p* = 0.04). Note that this effect is replicated also for the left angular gyrus (Figure S7D).

We reported the effect in the left precuneus because we found that changes in individuals’ neural-TD pattern similarity in the vertex condition to be associated positively with their TOJ memory performance in this key region (Figure 5C right image, Within-chapter condition: *r* = 0.60, *p* = 0.04; Across-chapter condition: *r* = 0.40, *p* = 0.02), implying these multivoxel representations for temporal distances are relevant neurobiological prerequisites for the ability to support memory retrieval.

**Figure 6:**
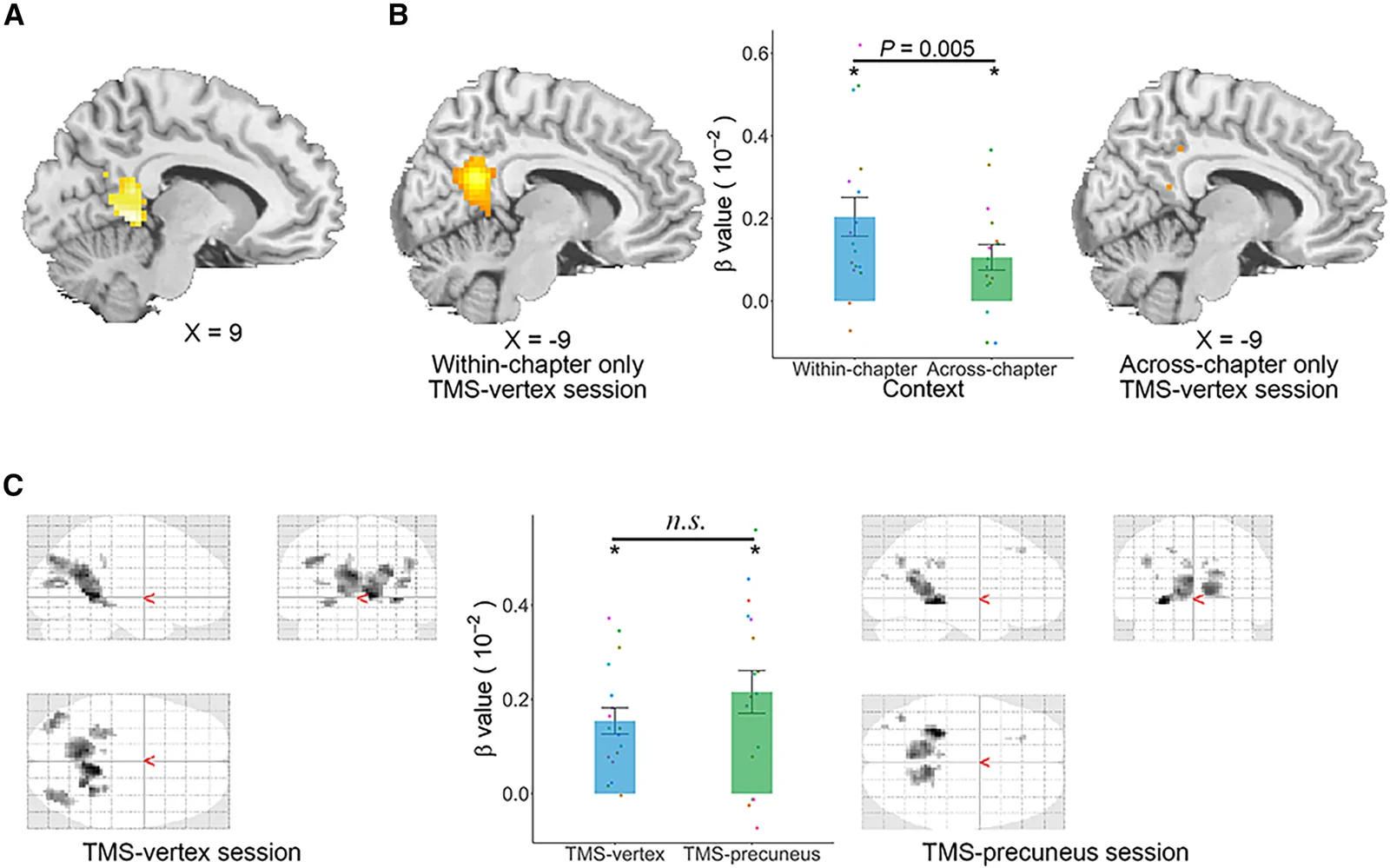
Univariate BOLD scaling with temporal distance is preserved after precuneus rTMS, dissociating from the attenuated multivoxel representation (A) Temporal distance (TD) and reaction time (RT) as modulatory regressors for TMS-vertex session (Within-chapter and Across-chapter trials collapsed). Having removed the influence of RT, neural activity in the precuneus/retrosplenial cortex and posterior hippocampus still increased with Log TD. (B) Log TD and RT as modulatory regressors separately for Within-chapter (left) and Across-chapter (right) conditions in the TMS-vertex session. After having removed the influence of RT, neural activity in the precuneus, extending into the retrosplenial cortex and posterior cingulate cortex, remained linearly associated with Log TD in the Within-chapter condition. The *β* values extracted from the cluster were also higher in the Within-chapter condition than in the Across-chapter condition (*p* = 0.005). (C) In this pmod result, in contrast to the removal of the multivoxel representation (Figure 5A), TMS to the precuneus did not significantly impact the activation intensity in the precuneus and retrosplenial cortex (little difference is observed when comparing the left image with the right image). Irrespective of TMS stimulation, activation in these clusters (containing the precuneus) significantly increased with temporal distance (middle image). Activation maps are displayed at *P*_uncorrected_ < 0.001. ∗ denotes *p* < 0.05, FDR-corrected, one-sample *t* test against zero. *n.s.* denotes not significant.

In the experimental condition where we disrupted the precuneal activity, a significant reduction in neural-behavioral correlation was also observed (Figure 5C right image; comparison between two correlations: *z* = 2.07, *p* = 0.04). This effect is also observed in the left hippocampus (see Figure S7B right image, Within-chapter condition: *r* = 0.59, *p* = 0.04; Across-chapter condition: *r* = 0.51, *p* = 0.08). We found that the parametric relationship in activation could not be attributed to difficulty by regressing out trial-by-trial reaction times (Figures 6A and 6B). Since the perturbation altered the mnemonic representation across parts of the PM system implicating the precuneus, the angular gyri, and the hippocampi, the putative disruption might have been effective through inducing alternation in functional connectivity between multiple regions, or more globally throughout the entire parietal memory network.^50^^,^^51^^,^^52^ In contrast, the activation-based Pmod analyses showed that TMS to the precuneus did not induce any discernible changes in the univariate BOLD intensity (Figure 6C).

## Discussion

The present study examined how temporal distances between episodic events are represented during memory retrieval. Using a naturalistic video-game paradigm, fMRI, RSA, and within-subject TMS, we found that retrieval trials involving similar temporal-distance magnitudes elicited similar multivoxel patterns in posterior medial cortex, including the precuneus and adjacent posterior cingulate/retrosplenial regions. This representational structure was stronger when events were drawn from the same chapter than from different chapters, indicating that temporal-distance coding is shaped by event context. Finally, perturbation of the medial parietal target attenuated the detectable multivoxel representation of temporal-distance magnitude while leaving univariate BOLD scaling intact, suggesting that posterior medial cortex contributes to, but is unlikely to solely determine, the retrieval of temporal relations in episodic memory.

Previous studies have associated neural similarity in the hippocampus with both spatial and temporal aspects of episodic memory in humans.^2^^,^^53^ Studies on rats have shown that gradually changing representations are manifested in the hippocampus.^12^ These studies have focused on neural similarities in the hippocampus,^27^^,^^46^^,^^54^ entorhinal cortex,^46^^,^^54^^,^^55^ and lateral prefrontal cortices,^27^^,^^46^^,^^54^^,^^56^ whereas presently we revealed a TD representation primarily in the PM system. Indeed, temporal representations are distributed across multiple brain regions, not solely encoded by the hippocampus.^18^^,^^57^ The parietal univariate and multivariate representation of temporal distances between pairs of episodic events underscore the significant contribution of parietal cortex,^58^^,^^59^ particularly the precuneus, to temporal aspects of memory. A recent study using mesoscale two-photon imaging in mice revealed that the retrosplenial cortex (RSC)—a region anatomically linked to the precuneus within the para-cingulate network definition—exhibits area-specific temporal encoding.^60^ Neurons in the mouse RSC displayed distinct, time-dependent sequential firing patterns that encoded both stimulus identity (“what”) and elapsed time (“when”). These findings position the precuneus, alongside the RSC and hippocampus, as part of a distributed network supporting the complex representation of time in memory. In the present context, this precuneal involvement might act in parallel with hippocampal cells that code specific moments in time or temporal positions,^8^^,^^61^^,^^62^^,^^63^ or function independently, contributing to a separate mnemonic establishment of episodes that complements the hippocampal memory system.^64^ Functional heterogeneity within the precuneus^65^^,^^66^^,^^67^ supports a mechanism where different subregions may contribute to temporal memory processing through scene construction and temporal organization of memory in concert with the hippocampus.^68^^,^^69^^,^^70^^,^^71^ Note that the present anatomical ROI analyses were not designed to resolve fine-grained functional subdivisions within the posterior medial cortex. The precuneus, posterior cingulate cortex, and retrosplenial cortex are anatomically adjacent and functionally interconnected, and the current spatial resolution and smoothing make sharp subregional attribution difficult. Future studies using higher-resolution imaging, individualized functional localizers,^72^ finer parcellation schemes such as the Neuromorphometrics or multimodal methods,^73^ or stimulation methods with high spatial resolution^74^ will be needed to determine whether temporal-distance representations are preferentially localized to the precuneus proper, PCC, retrosplenial cortex, or distributed across the PM memory network.

The neural representation of event-wise separation can also be interpreted with predictions by the Temporal Context Model,^17^ which stipulates that the memory information stored is stronger when paired images were associated in proximity (and even more so, within a shared context, such as our Within-Chapter trials). The influence of contextual similarity mirrors findings that narrative coherence can improve the recall of temporally distant events compared to when events are embedded in unrelated narratives.^75^^,^^76^^,^^77^^,^^78^^,^^79^ The precuneus may be important for binding events across distinct contexts, possibly together with other regions implicated in both temporal and spatial distance effects such as the intraparietal lobule and angular gyrus.^80^^,^^81^^,^^82^ Recent work, drawing upon context-resetting models,^83^ emphasizes the importance of resetting local event position codes in temporal order memory.^84^ The role of the precuneus and how it might be linked to hierarchical organization of nested neural events in long-term memory representations^29^^,^^85^^,^^86^ is worth further investigating.

Another key finding of the present study is that temporal distance representations were selectively expressed for events retrieved within a shared episodic context. Although contextual boundaries are known to influence episodic memory, our results extend this literature by demonstrating that context modulates the representational geometry of temporal distance itself. Rather than serving as a global metric of elapsed time, temporal distance appears to be represented within structured episodic frameworks, suggesting that temporal abstraction and contextual organization are jointly encoded during retrieval. This aligns with models proposing that posterior medial regions encode structured event relationships and integrate information across meaningful boundaries. Rather than representing time as a uniform scale, the precuneus may contribute to organizing temporal relationships within situational frameworks. These findings indicate that temporal distance is represented in a context-sensitive and relational manner rather than as an absolute, context-invariant metric.^16^

It is theoretically important that the multivariate representations were more vulnerable to magnetic stimulation than the voxel-wise aggregate signal intensity (TMS to the precuneus did not induce any discernible changes in univariate BOLD level,^87^ see Figure 6C). This differential effect caused by the TMS could be linked to the dissociation pattern seen behaviorally, where TMS to the precuneus significantly slowed down the retrieval speed (i.e., increased RT) but did not affect memory accuracy. One possibility is that the BOLD activation level in the precuneus is linked to memory accuracy. Indeed, neuronal synchronization in the macaque precuneus has been shown to reflect temporal order memory success.^88^ In contrast, the multivoxel pattern in the precuneus might carry information supporting memory search or evidence of accumulation speed, thus linking it to reaction times. These findings suggest that memory traces of temporal distances that are represented during retrieval could be conveyed in some localized multivoxel readouts housed in the PM system cortices, above and beyond the modulated changes in canonical BOLD activation.^89^ While rTMS significantly weakened the multivoxel representation of temporal distance in the precuneus, it did not produce a corresponding impairment in temporal order judgments.

Relatedly, the behavioral effects of TMS should be interpreted cautiously. The observed change in the context effect does not imply that the loss of temporal-distance neural structure improves temporal order memory. Rather, temporal order judgments may rely on multiple, partially separable sources of information,^1^ including graded temporal-distance representations, contextual familiarity, and chapter-level event structure. Perturbation of the target region may therefore have altered the balance among these sources, producing a behavioral context effect that need not map directly onto the RSA-derived temporal-distance representation. Because the present design cannot identify the retrieval strategy used on individual trials, this interpretation remains speculative. Future studies combining trial-wise strategy reports, sequential cueing, or computational modeling will be needed to determine how stimulation alters the decision processes underlying temporal order judgments. Moreover, the lack of a behavioral effect may reflect the brain’s capacity for rapid, compensatory reorganization. The brain regions involved in episodic memory form a highly interconnected network.^32^ It is plausible that upon temporary disruption of the precuneus,^90^^,^^91^ other nodes within the temporal memory network—such as the angular gyrus and middle frontal gyrus, where we also observed TD representations—were able to compensate to support successful performance. Indeed, while we targeted the precuneus, prior work indicates that TMS stimulation influences distributed networks, consistent with the notion of circuit-level effects.^92^ The observed changes in representational coding likely reflect perturbation of a broader posterior medial system supporting episodic organization. Building on connectivity findings between the hippocampus and neocortical regions^32^^,^^93^^,^^94^ and the hippocampal role in temporal context memory,^11^^,^^13^ our demonstration of a distributed pattern of temporal information implied a higher-level parietal mnemonic readout of temporal distances between episodic experiences. Accordingly, our findings should be interpreted as demonstrating that perturbation of the precuneus alters the expression of temporal-distance representations within the posterior medial memory network, rather than establishing that the precuneus is their sole locus.

Unlike paradigms using passive viewing (e.g., pictures, movies, narratives), our encoding phase involved active, first-person perspective gameplay, designed to create episodic memories more akin to real-life autobiographical experiences, which could differentially engage retrieval systems.^95^^,^^96^ Our findings dovetail with autobiographical memory (ABM) showing that the posteromedial network supports temporal organization across extended intervals. First, ABM ordering tasks reveal that accuracy increases with larger temporal separations and recruit precuneus/PCC and AG, with context-dependent vmPFC-PM connectivity.^97^ We observe a related pattern: TD codes are strongest for within-chapter pairs, consistent with ABM accounts that temporal organization is scaffolded by coherent situation models. Second, the scale-selective architecture of medial parietal cortex^98^ suggests a preferential role for longer, more abstract temporal ranges; our minutes-level TDs engage posteromedial regions, aligning with that gradient-based view. Third, a meta-analysis indicates a PCC-hippocampal axis for ABM with recent-remote differentiation.^99^ Our rTMS disruption of precuneal multivoxel TD code and the attenuation of precuneus-hippocampus pattern-behavior coupling is consistent with this axis supporting temporal structuring. Finally, a systematic review and meta-analysis emphasize complementary roles of the posteromedial cortex in constructing and elaborating episodic–autobiographical memories, via schema-supported temporal relations.^100^

Our hypothesis assumed that this code follows a logarithmic scale, based on prominent theories proposing that memory for time is psychologically compressed, akin to sensory perception.^2^ When we partially removed the respective influences of perceptual properties (e.g., SUFT model), the effects of TD representation in the posteromedial parietal cortex were largely unaffected (Figure S5J). However, while our model comparison confirmed that a Log TD model strongly explains the neural data, it also revealed that the raw TD model performs statistically equivalently. This suggests that while the precuneus represents temporal distance in a monotonic manner, the precise functional form of this representation—logarithmic versus other non-linear functions—remains an open question that our current design cannot definitively resolve. It is possible that the ∼18-min range of our study was not sufficiently large to reveal the compressive non-linearities that might dominate over longer, autobiographical timescales. Nevertheless, our truncation analyses robustly demonstrated that this temporal coding is a genuine gradient and not an artifact of comparing the extreme short and long ends of the temporal spectrum, lending support to the existence of a pattern-based mnemonic code in the precuneus.

Together, rather than representing elapsed time as a global metric, the posterior medial system appears to organize temporal relationships within structured episodic experiences. This perspective links temporal coding with theories of event segmentation and contextual memory organization, suggesting that temporal abstraction emerges from interactions between temporal and contextual representations within a distributed parietal network during retrieval of naturalistic first-person experience.

### Limitations of the study

Our sample of 17 participants, although comparable to previous within-subject TMS-fMRI studies of episodic memory,^52^^,^^87^ is modest by current standards. The counterbalanced within-subject crossover design, in which every participant completed both stimulation conditions using independent encoding materials, allows each participant to serve as their own control and thereby improves the precision of within-subject estimates; nevertheless, the present findings require replication in larger independent samples.

## Resource availability

### Lead contact

Requests for further information and resources should be directed to and will be fulfilled by the lead contact, Sze Chai Kwok (sze-chai.kwok@st-hughs.oxon.org).

### Materials availability

• This study did not generate new unique reagents.

### Data and code availability

• The data generated during the analysis are available at https://osf.io/4xf2k/. Raw data are available from the corresponding author upon request.
• All analyses of fMRI data were performed using MATLAB R2021a with standard functions and toolboxes. Behavioral data analyses were carried out using R 4.4.2, utilizing standard functions and packages. Codes for the mixed-effects models are available at https://osf.io/4xf2k/.
• Any additional information required to reanalyze the data reported in this paper is available from the lead contact upon request.

## Acknowledgments

This research is sponsored by the 10.13039/501100001809National Natural Science Foundation of China 32200912 (Q.Y.), 31872783 (Y.H.), and 32071060 (S.C.K.).

## Author contributions

Conceptualization, Q.Y. and S.C.K.; methodology, Q.Y., Y.K., K.A., and S.C.K.; investigation, Q.Y. and S.C.K.; writing – original draft, Q.Y.; writing – review and editing, Q.Y., Y.H., and S.C.K.; funding acquisition, S.C.K.; resources, Q.Y., K.A., Y.H., and S.C.K.; supervision, S.C.K.

## Declaration of interests

The authors declare no competing interests.

## Declaration of generative AI and AI-assisted technologies in the writing process

During the preparation of this work, the author(s) used ChatGPT in order to improve language readability. After using this tool or service, the author(s) reviewed and edited the content as needed and take(s) full responsibility for the content of the publication.

## STAR★Methods

### Key resources table

**Table:** 

REAGENT or RESOURCE	SOURCE	IDENTIFIER
**Software and algorithms**		

E-prime software	Psychology Software Tools, Inc., Pittsburgh, PA	N/A
SPM12	N/A	http://www.fil.ion.ucl.ac.uk/spm
RSA toolbox	N/A	http://www.mrc-cbu.cam.ac.uk/methods-and-resources/toolboxes/
MATLAB R2021a	N/A	https://www.mathworks.com

**Deposited data**		

Generated data and code	This Paper	OSF: https://osf.io/4xf2k/

**Other**		

Magstim Rapid2	The Magstim Company, Ltd., Whitland, UK	N/A
3-Tesla Siemens Trio magnetic resonance imaging scanner	Siemens Medical Solutions, Erlangen, Germany	N/A

### Experimental model and study participant details

Twenty individuals participated in the study (7 female and 13 male, 22.55 ± 1.54 years, mean ± sd). Data from 3 subjects were excluded due to either poor performance (1 subject performed at chance level) or scanner malfunction (projector crashed during scanning for 2 subjects at TMS-vertex session), resulting in a final group of 17 subjects (7 female and 10 male, 20.65 ± 1.54 years, mean ± sd). All subjects were unfamiliar with the video game, had normal or correct-to-normal vision and did not report neurological or psychiatric disorders or current use of psychoactive drugs. All subjects were eligible for MRI and TMS procedures based on standard MRI safety screening as well as on their answers to a TMS safety screening questionnaire.^101^ No subjects withdrew due to complication from the TMS or MRI procedures, and no negative treatment responses were observed. All subjects gave written informed consent and were compensated for their participation. All procedures were performed in accordance with the 1964 Helsinki declaration and its later amendments and approved by University Committee on Human Research Protection of East China Normal University (UCHRP-ECNU; protocol number: HR 023-2017). Our within-subjects (Table S1), counterbalanced design increases efficiency and reduces between-subjects variance. The final sample size is comparable to several previous within-subject TMS-fMRI studies of episodic memory,^52^^,^^87^ although we acknowledge that it is modest by current standards. Our counterbalanced within-subject crossover design, in which every participant completed both stimulation conditions using independent encoding materials, allows each participant to serve as his or her own control, thereby reducing between-subject variability and improving the precision of within-subject estimates. Nevertheless, the present findings should be interpreted with appropriate caution and will require replication in larger independent samples.

### Method details

#### Experimental design, stimuli, and tasks

For memory encoding, participants played an interactive video game containing seven distinct but related chapters, each in the range of tens of minutes on day 1 (Figure S1 and Table S1, see Video S1 for an excerpt of the encoding stage). By the nature of the video game, within-chapter segments contained more coherent narrative strands than those across chapters, yet all chapters were connected by a common plot. After a 24-hour retention period (day 2), on each trial, participants judged the temporal order of two images (extracted from their individually played video game, Figure 1B), depicting two time-points in their encoded memory, while their blood-oxygen-level-dependent (BOLD) activity was measured (TOJ task, Figure 1C). Assuming a scale-free temporal memory representation,^102^ we manipulated the between-images temporal distances (TD) for all pairs of images so that the TD distribution adhered to a power function permitting scale-invariance across subjects^103^ (60 levels of TD, Figure 1D). To test the interaction effect between TD and its encoding context, we manipulated the factor “context” by controlling whether the paired images presented at memory test were extracted from the same chapters or two adjacent chapters of the video game while keeping the 60 TDs fully matched between the two conditions (Within-chapter vs. Across-chapter, Figure 1E). The two-session design was required by the within-subjects TMS counterbalancing protocol. Each participant completed both the control vertex stimulation session and the precuneus stimulation session, allowing stimulation effects to be assessed within individuals. The two sessions also minimized carryover of low-frequency rTMS effects and ensured that each retrieval test was performed under a distinct stimulation condition.

Video S1. A movie excerpt from the encoding stage

##### Encoding: Interactive video game

The action-adventure video game (Beyond: Two Souls) was created by the French game developer Quantic Dream and played in the PlayStation 4 video game console developed by Sony Computer Entertainment. Participants played the game using a first-person perspective. To ensure that the participants mastered the operational capability, they were trained to play the game with two additional game chapters (Training chapters: Welcome to the CIA, and The Embassy). The training session varied in duration depending on the dexterity of each participant using the console (40 – 60 min per chapter). After the training session, participants played 14 chapters in total across two sessions: 7 in Experimental Session 1 and then another 7 in Session 2 (Figure 1). The video game they played were recorded and stored as a single video file in MP4 format (Chapters 1∼7: My Imaginary Friend, First Interview, First Night, Alone, The Experiment, Night Session, Hauntings; Chapters 8∼14: The Party, Like Other Girls, Separation, Old Friends, Norah, Agreement, Briefing; see Figure S1). To ensure for the best TMS effect, we have arranged the participants to undergo anatomical MRI scans, familiarize themselves with the gameplay, and have their active motor threshold measured (for TMS) *on a different day* prior to Day 1 when the experimental session proper began. This helped ensure the delay between TMS administration (outside MRI scanner) and the retrieval test (inside MRI scanner) was minimal.

##### Retrieval (scanned): Temporal order judgment (TOJ) task

Our question concerns the retrieval stage. We parametrized a large set of pairs of event-moments geometrically separated by varying temporal intervals and applied a multivariate searchlight representational similarity analysis (RSA)^39^ to compare neural representational dissimilarity matrices (RDM) with several parametric hypothetical/candidate models. Applied across the entire brain, the searchlight approach identifies local multi-voxel patterns at a voxel level within the size of the 9-mm radius spherical searchlight, giving us a snapshot of the locally distributed neural representation supporting temporal distances in episodic memory. In addition, to enhance the causal strength of the anatomical associations, we disrupted the identified critical region with repetitive transcranial magnetic stimulation (rTMS, Figure 1F). Although rTMS may influence broader neural circuits rather than solely a single spatially demarcated area,^92^ targeting this specific node allowed us to test its functional necessity in mediating the representation of temporal distances through reversible manipulation.

The TOJ retrieval task required participants to choose the image that happened earlier in the video game they had encoded. Operationally, TOJ tasks do not directly ask for temporal distance, but they rely on the brain’s representation of temporal structure, including both order and distance. Here, we varied the lag between events (60 levels of them, see below) and examined how brain activity and performance scale with that lag—effectively using the TOJ task as a proxy for temporal distance representation. We deem TOJ as a practical and controlled way to probe how time separation is organized and accessed during retrieval.

Since the encoding stage was entirely determined by individual participants, it would be impossible to make their judgment relying on inferred distance based on image properties.^104^ The task was administered inside an MRI scanner, where visual stimuli were presented using E-prime software (Psychology Software Tools, Inc., Pittsburgh, PA), as back-projected via a mirror system to the participant. Each trial was presented for 5 s during which participants performed the temporal order judgment. They were then allowed 3 s to report their confidence level following the memory judgement. Participants performed the TOJ task using their index and middle fingers of one of their hands via an MRI compatible five-button response keyboard (Sinorad, Shenzhen, China). Participants reported their confidence level (“Very Low”, “Low”, “High”, or “Very High”) regarding their own judgment of the correctness of TOJ with four fingers (thumb was not used) of the other hand. The left/right hand response contingency was counterbalanced across participants. Participants were instructed to respond as quickly and accurately as possible, ensuring that participants engaged in active memory retrieval processes. Participants were told they should report their confidence level in a relative way and make use of the whole confidence scale. Following these judgments, a fixation cross with a variable duration (1 – 6 s) was presented. Each participant completed 240 trials in each of the two experimental sessions. Participants were given 15 practice trials using images extracted from the two additional chapters they had played in the training session out of the scanner to ensure they understood the task procedure. Participants completed a surprise recognition test after TOJ task outside the scanner; data of which are not reported here.

For the TOJ task, we selected still images from the subject-specific recorded videos which the participants had played the day before. Each second in the video consisted of 29.97 static images (frames). For each game-playing session, 240 pairs of images were extracted from the seven chapters and were paired up for the task based on the following criteria: (1) the two images had to be extracted from either the same chapters or adjacent chapters (Within-chapter vs. Across-chapter); (2) the temporal distance (TD) between the two images were matched between Within- and Across-chapter condition; (3) in order to maximize the TD, we first selected the second longest chapter of the video and determined the longest TD according to a power function (power = 1.5), at the same time ensuring the shortest TD to be longer than 30 frames. We generated 60 progressive levels of TD among these pairs (each level repeated twice). In sum, three within-subjects factors regarding the TOJ retrieval task were manipulated: (1) 60 TD levels permitting scale-invariance across subjects between two images (see below); (2) Context (two images extracted from either Within- or Across-chapter); (3) TMS stimulation (TMS-precuneus vs. TMS-vertex, see below).

##### Selection of 60 levels of temporal distances (TDs)

To maximize the range of all TDs, we first selected the second longest chapter of the video game and determined the longest TD (*L*), while ensuring the shortest TD to be longer than 30 frames. The 60 TD levels were selected according to this function,

$${TD}_{n}=L*{\left(\frac{n}{60}\right)}^{1.5}$$

where *L* denotes duration of the second longest chapter of the video game in each experimental session, *n* denotes TD level, and values of *TD*_*n*_ were rounded to the nearest integer using the “round” function in MATLAB. Note that the actual TDs were different across participants, but since we applied a power function, the scale was thus rendered invariant.^102^ Image-pairs extraction from each of the chapters were independently conducted across participants, but the lengths of each chapter played by participants are comparable across them. The numbers of image-pairs extracted from each of the chapters were approximately equal within subjects. Across all participants, the shortest and the longest TD are 0.90 s (∼1/60 minute) and 1075.51 s (∼17.93 minutes) respectively.

#### Transcranial magnetic stimulation

##### TMS procedure and protocol

TMS were applied using a 70 mm Double Air Film Coil connected to a Magstim Rapid2 (The Magstim Company, Ltd., Whitland, UK). To localize the target brain regions precisely, we obtained individual anatomical T1-weighted magnetic resonance images and then imported them into BrainSight (Rogue Research Inc., Montreal, Canada) for stereotaxic registration of the TMS coil with the participants’ brain. The position of the coil and the subject’s head were co-registered with BrainSight and monitored using a Polaris Optical Tracking System (Northern Digital, Waterloo, Canada) during TMS. Positional data for both rigid bodies were registered in real time to a common frame of reference and were superimposed onto the reconstructed three-dimensional MRI images of the subject using BrainSight. The center of the coil was continuously monitored to be directly over the site of interest. For all sites (vertex, precuneus, and motor areas for measuring resting motor threshold), the TMS coil was held tangential to the surface of the skull and was placed in a rostro-caudal direction. An adjustable frame was used to hold the TMS coil firmly in place, while the participants rested their heads on the chin rest. Head movements were monitored constantly by BrainSight and were negligible. We measured subjects’ resting motor threshold, defined as the lowest TMS intensity delivered over the motor cortex necessary to elicit visible twitches of the right index finger in at least 5 out of 10 consecutive pulses. The location used to determine the resting threshold was identified with a single pulse of TMS over the motor cortex at the left hemisphere. The TMS coil was systematically moved until the optimal cortical site was located to induce the largest and most reliable motor response; this stimulus output was then recorded. The TMS intensity was then calibrated at 110% of individual resting threshold (stimulator output: 75.2 ± 6.9%, mean ± se, range from 63% to 88%, Table S1). In Experimental Session 1 and 2, as the TMS was applied at a low-frequency rate of 1 Hz with an uninterrupted duration of 20 min. We conducted the TMS manipulation immediately before we sent the participants into the scanner for the retrieval test. Low-frequency stimulation (1 Hz) imposes intracortical inhibitory effects, and the duration of the inhibitory after-effects varies with the length of the stimulation such that longer stimulation duration would induce a longer efficacy period. The rTMS protocol adopted in our study is known to induce >60 min inhibitory effects.^101^^,^^105^^,^^106^ These inhibitory effects are also known to level off within hours by the end of the stimulation. Given that our TOJ retrieval test lasted <45 min, the duration of rTMS effects should have been long-lasting enough for the tasks.

##### TMS stimulation sites

The target stimulation was delivered to the precuneus^107^ (MNI x, y, z = 6, -70, 44), whereas the control stimulation was delivered to the vertex. For neuronavigation, the target MNI coordinate was reverse-normalized into each individual’s native T1 anatomical space using the inverse of the spatial normalization parameters estimated during MRI preprocessing in SPM12. The resulting subject-specific target coordinate was then imported into BrainSight and superimposed onto the participant’s reconstructed three-dimensional T1 image. The TMS coil was co-registered with BrainSight using a Polaris Optical Tracking System and the coil center was continuously monitored and maintained directly over the individual-space target coordinate throughout the stimulation session. The vertex was defined individually by the point of the same distance to the left and the right pre-auricular, and of the same distance to the nasion and the inion. Due to the folding of the two cerebral hemispheres, the stimulated vertex site lies at a considerable distance from the TMS coil, thereby diminishing the effectiveness of the magnetic pulses. Stimulating the vertex is not known to produce any memory task-relevant effects and deemed a reliable control site. Stimulation magnitude and protocols in the present study were validated in similar studies that produce memory-related changes by targeting at the precuneus^108^^,^^109^^,^^110^ or lateral parietal cortices^50^^,^^51^^,^^52^ with little effects on adjacent regions. Although our choice was of a left-lateralized stimulation coordinate (x = 6) and that the SPM cluster labeling uses “left” or “right” tags for univariate peaks, the TMS target itself was essentially medial (small value on the x coordinate), chosen in consistency with our prior work.^107^ We should note that medial targets with small MNI x offsets are commonplace in TMS studies due to skull geometry (scalp-to-brain distance) and coil placement constraints, while still engaging the midline (medial) tissue. Immediately after the end of the stimulation, participants performed four runs of temporal order judgment task in the MRI scanner (delay period between the end of TMS and the beginning of MRI: *M*_precuneus_ = 15.29 min, *M*_vertex_ = 20.76 min, *t* (16) = -0.87, *p* = 0.4).

### Quantification and statistical analysis

#### MRI data acquisition and preprocessing

##### Data acquisition

All the participants were scanned in a 3-Tesla Siemens Trio magnetic resonance imaging scanner using a 32-channel head coil (Siemens Medical Solutions, Erlangen, Germany) at ECNU. In each of the two experimental sessions, a total of 1,350 fMRI volumes were acquired for each subject across 4 runs. The functional images were acquired with the following sequence: TR = 2000 ms, TE = 30 ms, field of view (FOV) = 230 × 230 mm, flip angle = 70°, voxel size = 3.6 × 3.6 × 4 mm, 33 slices, scan orientation parallel to AC-PC plane. High-resolution T1-weighted MPRAGE anatomy images were also acquired (TR = 2530 ms, TE = 2.34 ms, TI = 1100 ms, flip angle = 7°, FOV = 256 × 256 mm, 192 sagittal slices, 0.9 mm thickness, voxel size = 1 × 1 × 1 mm).

##### Preprocessing

Preprocessing was conducted using SPM12 (http://www.fil.ion.ucl.ac.uk/spm). Scans were realigned to the middle EPI image. The structural image was co-registered to the mean functional image, and the parameters from the segmentation of the structural image were used to normalize the functional images that were resampled to 3 × 3 × 3 mm. The realigned normalized images were then smoothed with a Gaussian kernel of 8-mm full-width half maximum (FWHM) to conform to the assumptions of random field theory and improve sensitivity for group analyses.^111^ Data were analyzed using general linear models and representational similarity analyses as described below with a high-pass filter cutoff of 256 s and autoregressive AR(1) model correction for autocorrelation.

#### Functional MRI data analysis

##### Parametric modulation analysis

First-level models were performed on the fMRI data collected from the TMS-vertex session only (either all the trials altogether or separately for Across-chapter vs. Within-chapter conditions). In all models, each of the 240 trials was modeled with a canonical hemodynamic response function as an event-related response with a duration of 5 s.

For the TMS-vertex session (Across-chapter and Within-chapter trials collapsed), we performed two parametric modulation analyses (pmod), each with a different combination of modulatory regressor/regressors (namely, TD; TD + RT). For the TD pmod, we assigned the actual TD values at encoding as the modulatory parameter and used the polynomial function up to first order. Several regressors of no interest were also included: 6 head movement regressors and 1 missing trial regressor (i.e., no-response trials; number of missing trials of Across-chapter condition: 5.65 ± 6.96 of Within-chapter condition: 5.29 ± 6.8; *n* = 17, mean ± sd) and the run mean. The purpose of this analysis was to test any linear TD-dependent modulation of signal intensity in the brain between the TD between the two images at encoding and the brain activity during TOJ retrieval of the same events. For the TD + RT pmod, we aimed to identify the voxels whose activities changed as a function of TD after the removal of the influence of reaction times. Each subject’s RTs corresponding to each TD level were entered as the modulatory parameter, together with the regressors of no interest as above.

For the Across-chapter vs. Within-chapter comparison, we also performed two pmod analyses with identical sets of regressors as described above (namely, TD; TD + RT). We looked for changes in brain responses as a linear function of the regressor of interest (i.e., TD). Maps were created by multiple regression analyses between the observed signals and regressors. The contrast maps from the first-level model of parametric analyses were taken for second-level group analyses and entered one-sample *t*-tests. The group analyses were performed for each contrast using a random effects model.^112^ The statistical threshold was set at *p* < 0.05 (FWE corrected) at cluster level and *p* < 0.001 at an uncorrected peak level according to the SPM12 standard procedure. The activation cluster locations were indicated by the peak voxels on the normalized structural images and labeled using the nomenclature of Talairach and Tournoux.^113^

##### Searchlight representational similarity analysis (searchlight RSA)

RSA was conducted using the RSA toolbox (http://www.mrc-cbu.cam.ac.uk/methods-and-resources/toolboxes/) on the fMRI data following realignment and normalization, but without smoothing.

In the Across-chapter vs. Within-chapter comparison, each unique TD level was modeled with a separate regressor and was contrasted to produce a T-statistic map (spmT maps), creating 120 statistical maps in total (Across- vs. Within-chapter conditions; 2 repetitions for each TD level). For the TMS-vertex vs. TMS-precuneus comparison, we collapsed the respective trials within the Across- and Within-chapter conditions and generated 60 statistical maps in either of the two sessions (4 repetitions for each TD level). Using searchlight RSA, spherical searchlights with a radius of 9 mm (93 voxels, volume = 2,511 mm^3^) were extracted from the brain volume and then the data (i.e., signal intensity) for the 60 TD levels were Person product-moment (1 - *r*) correlated with every other level to generate a representational dissimilarity matrix (RDM), reflecting the between-condition dissimilarity of BOLD signal response. These neural RDMs were then Spearman-rank correlated with a set of candidate RDMs (Figure 3F), reflecting different predictions of the information carried by similarity structure of neural signal responses and generated correlational maps (r-maps). Finally, these *r*-maps were converted to *z*-maps using Fisher transformation. All the *z*-maps were then submitted to a group-level one-sample *t*-test to identify voxels in which the similarity between the predicted RDM and observed neural RDM was greater than zero. This allowed us to identify voxels in which information of TD at retrieval might be represented (Figure S3). The statistical threshold was set as identical to those employed in the univariate analysis, which was at *p* < 0.05 (FWE corrected) at cluster level and *p* < 0.001 at an uncorrected peak level. It is worth noting that RSA does not require visual similarity between empirical and model RDMs but similarity in the *rank order* of pairwise distances (i.e., systematic covariation). The logic of RSA is that if a candidate model captures the information represented in a brain region, the rank order of pairwise dissimilarities in the neural data should correlate with the model’s predicted dissimilarities.^39^^,^^40^ Moreover, the group averaged neural RDM does not necessarily fully reflect individual variability in their subject-level neural RDMs. Our searchlight RSA here quantifies whether the geometry of neural dissimilarities matches the geometry predicted by a model. The inference does not rely on absolute values within the group-average RDM, but on correspondence between participant-specific neural RDMs and the model RDM. The resulting neural RDM tested whether trials involving similar temporal-distance magnitudes elicited similar multivoxel patterns. This analysis should be interpreted as a representation of retrieved temporal-distance magnitude, rather than as a direct item-to-item similarity measure between the two probed events.

##### Leave-one-subject-out approach (LOSO), functional, and anatomical ROIs

We applied a LOSO approach to create functional ROIs to avoid statistical bias (Esterman et al., 2010). For instance, to identify a ROI (i.e., conjunction mask in Figure 3D) for Subj01, we estimated the contrast using a one-sample *t*-test on the whole-brain searchlight z-maps obtained from Subj02 to Subj17. Likewise, we also estimated the contrast using a one-sample *t*-test on the contrast maps obtained from the Pmod analysis of Subj02 to Subj17. We set the same threshold reported above to extract clusters from these two statistical maps. We then overlaid the two resultant maps and extracted a conjunction region (mask01), with which we used to extract the value in the searchlight *z*-map from Subj01 for further statistical analysis. This procedure was repeated 17 times and generated 17 different ROIs, which provided statistically independent regions to extract values for testing differences between conditions.

For the anatomical ROIs (depicted in Figures 5 and S7), 7 regions (bilateral precuneus, bilateral angular gyrus, bilateral hippocampus and occipital cortex) of AAL template^114^ were created as masks. We extracted and averaged the similarity value within these masks for each subject for statistical tests. For this analysis, for consistency across all candidate ROIs, we opted to use anatomical ROI (rather than functional ROI obtained from Figure 3D) for the precuneus. For ROI-based analyses and RSA model comparisons involving multiple candidate models or regions, *p* values were corrected using the FDR procedure across the corresponding family of tests. FWE cluster-level correction is standard for whole-brain voxel-wise SPM analyses given the large, spatially structured number of comparisons, whereas FDR is appropriate for the smaller, discrete family of RSA model/ROI comparisons, following Nili et al.^39^

#### Candidate representational dissimilarity matrices (RDM)

*Model 0* (Raw TD RDM, 60 × 60) provided a comparison model that did not assume logarithmic compression. “Raw TD” refers to an RDM constructed directly from the untransformed temporal-distance values, rather than the empirical neural data itself. For this model, we computed the absolute difference directly from the subject-specified TD values (in seconds) for each pair of images without applying any log transformation. These raw temporal differences were calculated pairwise across all 60 TD levels to produce 60 × 59/2 values, which were then assigned to the corresponding cells of the RDM.

*Model 1* (Log TD RDM, 60 × 60): We ranked the difference across the 60 TD levels, from the shortest to the longest TD, for the Within-chapter condition (or Across-chapter condition; the two RDMs were identical because of their matched TD). We first log-transformed the subject-specified TD values for each pair of images and then computed the differences with and among every other TD level producing 60 × 59/2 values, which were then assigned to the corresponding cells of the RDM.

*Model 0* and *Model 1* model for *second-order similarity* or distances between neural patterns across trials (i.e., dissimilarity between neural patterns elicited by different TD conditions)—not the perceptual similarity between the two images presented on a single trial. They encode hypothesized representational structure along the temporal dimension independent of stimulus similarity: We are testing whether the fMRI neural pattern during TD retrieval varies as a function of the magnitude of temporal separation being retrieved. Our prediction is that trials with similar temporal distances (e.g., TD = 5 s vs. TD = 7 s) should elicit similar neural patterns (low dissimilarity in the RDM), because the brain is performing a similar mnemonic operation—retrieving two closely spaced moments. Conversely, trials with very different temporal distances (e.g., TD = 5 s vs. TD = 500 s) should elicit rather dissimilar neural patterns (high dissimilarity in the RDM), because the retrieval operations differ substantially due to the enormous temporal distances embedded in the trial.

*Model 2* (Situational Change RDM, 60 × 60): Since the temporal and spatial dimensions were closely inter-correlated. We checked whether the situational change might influence the neural patterns in those voxels that represent the TD information. We analyzed the subject-specific videos frame by frame and marked out the boundaries at which a situational location had changed (see illustration in Figure S1D). Then we computed the numbers of situational changes contained in each of the paired images and then computed the differences with and among every other condition producing 60 × 59/2 values, which were then assigned to the corresponding cells of the RDM.

Details for eight additional candidate models (perceptual: RGB cross-correlation, RGB intensity, RGB histogram, SIFT, SURF, HOG; behavioral: Reaction time, accuracy) are described in the Text S2.

#### Behavioral data analysis

Participants’ accuracy and reaction times (RTs) in the temporal order judgment task were analyzed using mixed-effects models in R. Accuracy was modeled using a mixed-effects logistic regression, while RTs were analyzed with a linear mixed-effects model. For both models, centered temporal distances (TD; unit in frames), Context (Across-chapter vs. Within-chapter), and TMS session (TMS-vertex vs. TMS-precuneus) were included as fixed effects, along with all two- and three-way interactions. A random intercept for Subject was included in both models to account for individual variability. The logistic regression model for accuracy used a binomial distribution and likelihood ratio tests (LRT) for significance testing. The linear mixed-effects model for RT employed Kenward-Roger approximation for degrees of freedom. The *afex* package^115^ was employed for model fitting. For both accuracy and RT, significant interactions were followed up with simple effects analyses using estimated marginal means (*emmeans* package in R) and pairwise contrasts to identify specific differences between conditions. Where appropriate, post-hoc pairwise comparisons were conducted for multiple comparisons.

## References

[1] Friedman W.J. (1993). Memory for the time of past events. Psychol. Bull..

[2] Howard M.W. (2018). Memory as perception of the past: Compressed time in mind and brain. Trends Cogn. Sci..

[3] Buhusi C.V., Meck W.H. (2005). What makes us tick? Functional and neural mechanisms of interval timing. Nat. Rev. Neurosci..

[4] Meck W.H., Penney T.B., Pouthas V. (2008). Cortico-striatal representation of time in animals and humans. Curr. Opin. Neurobiol..

[5] Mauk M.D., Buonomano D.V. (2004). The neural basis of temporal processing. Annu. Rev. Neurosci..

[6] Jin D.Z., Fujii N., Graybiel A.M. (2009). Neural representation of time in cortico-basal ganglia circuits. Proc. Natl. Acad. Sci. USA.

[7] Leon M.I., Shadlen M.N. (2003). Representation of time by neurons in the posterior parietal cortex of the macaque. Neuron.

[8] Naya Y., Suzuki W.A. (2011). Integrating what and when across the primate medial temporal lobe. Science.

[9] Tsao A., Sugar J., Lu L., Wang C., Knierim J.J., Moser M.B., Moser E.I. (2018). Integrating time from experience in the lateral entorhinal cortex. Nature.

[10] McGaugh J.L. (2000). Memory--a century of consolidation. Science.

[11] Ezzyat Y., Davachi L. (2014). Similarity breeds proximity: pattern similarity within and across contexts is related to later mnemonic judgments of temporal proximity. Neuron.

[12] Manns J.R., Howard M.W., Eichenbaum H. (2007). Gradual changes in hippocampal activity support remembering the order of events. Neuron.

[13] Hsieh L.T., Gruber M.J., Jenkins L.J., Ranganath C. (2014). Hippocampal activity patterns carry information about objects in temporal context. Neuron.

[14] Nielson D.M., Smith T.A., Sreekumar V., Dennis S., Sederberg P.B. (2015). Human hippocampus represents space and time during retrieval of real-world memories. Proc. Natl. Acad. Sci. USA.

[15] Rubin A., Geva N., Sheintuch L., Ziv Y. (2015). Hippocampal ensemble dynamics timestamp events in long-term memory. eLife.

[16] Foudil S.A., Kwok S.C., Macaluso E. (2020). Context-Dependent Coding of Temporal Distance Between Cinematic Events in the Human Precuneus. J. Neurosci..

[17] Polyn S.M., Norman K.A., Kahana M.J. (2009). A context maintenance and retrieval model of organizational processes in free recall. Psychol. Rev..

[18] Tsao A., Yousefzadeh S.A., Meck W.H., Moser M.B., Moser E.I. (2022). The neural bases for timing of durations. Nat. Rev. Neurosci..

[19] Umbach G., Kantak P., Jacobs J., Kahana M., Pfeiffer B.E., Sperling M., Lega B. (2020). Time cells in the human hippocampus and entorhinal cortex support episodic memory. Proc. Natl. Acad. Sci. USA.

[20] Milivojevic B., Varadinov M., Vicente Grabovetsky A., Collin S.H.P., Doeller C.F. (2016). Coding of Event Nodes and Narrative Context in the Hippocampus. J. Neurosci..

[21] Ben-Yakov A., Henson R.N. (2018). The Hippocampal Film Editor: Sensitivity and Specificity to Event Boundaries in Continuous Experience. J. Neurosci..

[22] DuBrow S., Davachi L. (2016). Temporal binding within and across events. Neurobiol. Learn. Mem..

[23] Zheng J., Schjetnan A.G.P., Yebra M., Gomes B.A., Mosher C.P., Kalia S.K., Valiante T.A., Mamelak A.N., Kreiman G., Rutishauser U. (2022). Neurons detect cognitive boundaries to structure episodic memories in humans. Nat. Neurosci..

[24] Michelmann S., Staresina B.P., Bowman H., Hanslmayr S. (2018). Speed of time-compressed forward replay flexibly changes in human episodic memory. Nat. Hum. Behav..

[25] Zuo S., Wang L., Shin J.H., Cai Y., Zhang B., Lee S.W., Appiah K., Zhou Y.D., Kwok S.C. (2020). Behavioral evidence for memory replay of video episodes in the macaque. eLife.

[26] Jenkins L.J., Ranganath C. (2010). Prefrontal and medial temporal lobe activity at encoding predicts temporal context memory. J. Neurosci..

[27] Lositsky O., Chen J., Toker D., Honey C.J., Shvartsman M., Poppenk J.L., Hasson U., Norman K.A. (2016). Neural pattern change during encoding of a narrative predicts retrospective duration estimates. eLife.

[28] Zhu X., Jiang H., Li C., Pan K., Santos-Pata D., Zhang S., Kwok S.C. (2025). Replay of factorized temporal journey. Research Square.

[29] Baldassano C., Chen J., Zadbood A., Pillow J.W., Hasson U., Norman K.A. (2017). Discovering Event Structure in Continuous Narrative Perception and Memory. Neuron.

[30] Chen J., Leong Y.C., Honey C.J., Yong C.H., Norman K.A., Hasson U. (2017). Shared memories reveal shared structure in neural activity across individuals. Nat. Neurosci..

[31] Honey C.J., Thesen T., Donner T.H., Silbert L.J., Carlson C.E., Devinsky O., Doyle W.K., Rubin N., Heeger D.J., Hasson U. (2012). Slow cortical dynamics and the accumulation of information over long timescales. Neuron.

[32] Ranganath C., Ritchey M. (2012). Two cortical systems for memory-guided behaviour. Nat. Rev. Neurosci..

[33] Brunec I.K., Robin J., Olsen R.K., Moscovitch M., Barense M.D. (2020). Integration and differentiation of hippocampal memory traces. Neurosci. Biobehav. Rev..

[34] Yassa M.A., Stark C.E.L. (2011). Pattern separation in the hippocampus. Trends Neurosci..

[35] Favila S.E., Chanales A.J.H., Kuhl B.A. (2016). Experience-dependent hippocampal pattern differentiation prevents interference during subsequent learning. Nat. Commun..

[36] Chanales A.J.H., Oza A., Favila S.E., Kuhl B.A. (2017). Overlap among Spatial Memories Triggers Repulsion of Hippocampal Representations. Curr. Biol..

[37] Zacks J.M., Speer N.K., Swallow K.M., Braver T.S., Reynolds J.R. (2007). Event perception: a mind-brain perspective. Psychol. Bull..

[38] Wen T., Egner T. (2022). Retrieval context determines whether event boundaries impair or enhance temporal order memory. Cognition.

[39] Nili H., Wingfield C., Walther A., Su L., Marslen-Wilson W., Kriegeskorte N. (2014). A toolbox for representational similarity analysis. PLoS Comput. Biol..

[40] Kriegeskorte N., Mur M., Bandettini P. (2008). Representational similarity analysis - connecting the branches of systems neuroscience. Front. Syst. Neurosci..

[41] Greene M.R., Baldassano C., Esteva A., Beck D.M., Fei-Fei L. (2016). Visual scenes are categorized by function. J. Exp. Psychol. Gen..

[42] Aminoff E.M., Toneva M., Shrivastava A., Chen X., Misra I., Gupta A., Tarr M.J. (2015). Applying artificial vision models to human scene understanding. Front Comput Neurosc.

[43] Lowe D.G. (2004). Distinctive Image Features from Scale-Invariant Keypoints. Int. J. Comput. Vis..

[44] Bay H., Ess A., Tuytelaars T., Van Gool L. (2008). Speeded-Up Robust Features (SURF). Comput. Vis. Image Underst..

[45] Dalal N., Triggs B. (2005).

[46] Deuker L., Bellmund J.L., Navarro Schröder T., Doeller C.F. (2016). An event map of memory space in the hippocampus. eLife.

[47] Bright I.M., Meister M.L.R., Cruzado N.A., Tiganj Z., Buffalo E.A., Howard M.W. (2020). A temporal record of the past with a spectrum of time constants in the monkey entorhinal cortex. Proc. Natl. Acad. Sci. USA.

[48] Nelson S.M., McDermott K.B., Petersen S.E. (2012). In favor of a 'fractionation' view of ventral parietal cortex: comment on Cabeza et al. Trends Cogn. Sci..

[49] Richter F.R., Cooper R.A., Bays P.M., Simons J.S. (2016). Distinct neural mechanisms underlie the success, precision, and vividness of episodic memory. eLife.

[50] Eldaief M.C., Halko M.A., Buckner R.L., Pascual-Leone A. (2011). Transcranial magnetic stimulation modulates the brain's intrinsic activity in a frequency-dependent manner. Proc. Natl. Acad. Sci. USA.

[51] Nilakantan A.S., Bridge D.J., Gagnon E.P., VanHaerents S.A., Voss J.L. (2017). Stimulation of the Posterior Cortical-Hippocampal Network Enhances Precision of Memory Recollection. Curr. Biol..

[52] Wang J.X., Rogers L.M., Gross E.Z., Ryals A.J., Dokucu M.E., Brandstatt K.L., Hermiller M.S., Voss J.L. (2014). Targeted enhancement of cortical-hippocampal brain networks and associative memory. Science.

[53] Liu Y., Howard M.W. (2020). Generation of Scale-Invariant Sequential Activity in Linear Recurrent Networks. Neural Comput..

[54] Copara M.S., Hassan A.S., Kyle C.T., Libby L.A., Ranganath C., Ekstrom A.D. (2014). Complementary roles of human hippocampal subregions during retrieval of spatiotemporal context. J. Neurosci..

[55] Kyle C.T., Smuda D.N., Hassan A.S., Ekstrom A.D. (2015). Roles of human hippocampal subfields in retrieval of spatial and temporal context. Behav. Brain Res..

[56] Tiganj Z., Cromer J.A., Roy J.E., Miller E.K., Howard M.W. (2018). Compressed Timeline of Recent Experience in Monkey Lateral Prefrontal Cortex. J. Cogn. Neurosci..

[57] Kwok S.C., Lapate R.C., Sakon J.J., van Wassenhove V., Xue G., Zheng J. (2025). Neural representation of episodic time. J. Neurosci..

[58] Gilmore A.W., Nelson S.M., McDermott K.B. (2015). A parietal memory network revealed by multiple MRI methods. Trends Cogn. Sci..

[59] Wagner A.D., Shannon B.J., Kahn I., Buckner R.L. (2005). Parietal lobe contributions to episodic memory retrieval. Trends Cogn. Sci..

[60] Garvert A.C., Bieler M., Witoelar A., Vervaeke K. (2025). Area-specific encoding of temporal information in the neocortex. Cell Rep..

[61] Eichenbaum H. (2014). Time cells in the hippocampus: a new dimension for mapping memories. Nat. Rev. Neurosci..

[62] Pan K., Guo X., Zheng Z., Zhu Z., Wu H., Zhu J., Ye H., Cai Y., Santos-Pata D., Zhang J. (2024). Components of Mnemonic Metacognition Constitutionally Supported by High Gamma Activity Between the Precuneus and Hippocampus. bioRxiv.

[63] Sakon J.J., Naya Y., Wirth S., Suzuki W.A. (2014). Context-dependent incremental timing cells in the primate hippocampus. Proc. Natl. Acad. Sci. USA.

[64] Brodt S., Pöhlchen D., Flanagin V.L., Glasauer S., Gais S., Schönauer M. (2016). Rapid and independent memory formation in the parietal cortex. Proc. Natl. Acad. Sci. USA.

[65] Cavanna A.E., Trimble M.R. (2006). The precuneus: a review of its functional anatomy and behavioural correlates. Brain.

[66] Dadario N.B., Sughrue M.E. (2023). The functional role of the precuneus. Brain.

[67] Margulies D.S., Vincent J.L., Kelly C., Lohmann G., Uddin L.Q., Biswal B.B., Villringer A., Castellanos F.X., Milham M.P., Petrides M. (2009). Precuneus shares intrinsic functional architecture in humans and monkeys. Proc. Natl. Acad. Sci. USA.

[68] Barnett A.J., O'Neil E.B., Watson H.C., Lee A.C.H. (2014). The human hippocampus is sensitive to the durations of events and intervals within a sequence. Neuropsychologia.

[69] Hwang M.J., Lee S.A. (2024). Scene construction processes in the anterior hippocampus during temporal episodic memory retrieval. Hippocampus.

[70] Ye Q., Fidalgo C., Byrne P., Muñoz L.E., Cant J.S., Lee A.C.H. (2024). Using imagination and the contents of memory to create new scene and object representations: A functional MRI Study. Neuropsychologia.

[71] Palombo D.J., Verfaellie M. (2017). Hippocampal contributions to memory for time: evidence from neuropsychological studies. Curr. Opin. Behav. Sci..

[72] Russ B.E., Petkov C.I., Kwok S.C., Zhu Q., Belin P., Vanduffel W., Hamed S.B. (2021). Common functional localizers to enhance NHP & cross-species neuroscience imaging research. Neuroimage.

[73] Zhou X., Wang L., Tam S.K.E., Ortiz-Rios M., Yin D., Kwok S.C. (2026). Context-dependent functional diversity of dorsomedial posterior parietal neurons revealed by single-unit fMRI mapping during naturalistic viewing. bioRxiv.

[74] Zhang Y., Ren L., Liu K., Tong S., Yuan T.-F., Sun J. (2021). Transcranial ultrasound stimulation of the human motor cortex. iScience.

[75] Cohn-Sheehy B.I., Delarazan A.I., Crivelli-Decker J.E., Reagh Z.M., Mundada N.S., Yonelinas A.P., Zacks J.M., Ranganath C. (2022). Narratives bridge the divide between distant events in episodic memory. Mem. Cognit..

[76] Cohn-Sheehy B.I., Delarazan A.I., Reagh Z.M., Crivelli-Decker J.E., Kim K., Barnett A.J., Zacks J.M., Ranganath C. (2021). The hippocampus constructs narrative memories across distant events. Curr. Biol..

[77] Xia L.X., Liu K.G., Li X.Y., Ye Q. (2025). Encoding types and narrative coherence modulate the impact of emotions on temporal order memory. Acta Psychologica Sinica.

[78] Ye Q., Ke C., Wu Y., Cao Z. (2025). Distorted temporal position memory under negative emotional states. Cogn. Emot..

[79] Ye Q., Ke C. (2026). Emotion enhances relative but not absolute temporal memory: A meta-analysis. Psychon. Bull. Rev..

[80] Gauthier B., van Wassenhove V. (2016). Time Is Not Space: Core Computations and Domain-Specific Networks for Mental Travels. J. Neurosci..

[81] Gauthier B., Pestke K., van Wassenhove V. (2019). Building the Arrow of Time . Over Time: A Sequence of Brain Activity Mapping Imagined Events in Time and Space. Cereb. Cortex.

[82] Parkinson C., Liu S., Wheatley T. (2014). A common cortical metric for spatial, temporal, and social distance. J. Neurosci..

[83] Pu Y., Kong X.Z., Ranganath C., Melloni L. (2022). Event boundaries shape temporal organization of memory by resetting temporal context. Nat. Commun..

[84] Peng X., Cao Y., Sheng J., Zhou Y., Hu H., Xue G. (2025). A position coding model that accounts for the effects of event boundaries on temporal order memory. Cogn. Psychol..

[85] Hasson U., Chen J., Honey C.J. (2015). Hierarchical process memory: memory as an integral component of information processing. Trends Cogn. Sci..

[86] Ye Q., Zou F., Lau H., Hu Y., Kwok S.C. (2018). Causal evidence for mnemonic metacognition in human precuneus. J. Neurosci..

[87] Rafiei F., Rahnev D. (2022). TMS Does Not Increase BOLD Activity at the Site of Stimulation: A Review of All Concurrent TMS-fMRI Studies. eNeuro.

[88] Zuo S., Wang C., Wang L., Jin Z., Zhou X., Su N., Liu J., McHugh T.J., Kusunoki M., Kwok S.C. (2026). Neural population dynamics and temporal context cells in macaque medial parietal cortex support temporal order memory. PLoS Biol..

[89] Kwok S.C., Macaluso E. (2015). Immediate memory for “when, where and what”: Short-delay retrieval using dynamic naturalistic material. Hum. Brain Mapp..

[90] Ye Q., Zou F., Dayan M., Lau H., Hu Y., Kwok S.C. (2019). Individual susceptibility to TMS affirms the precuneal role in meta-memory upon recollection. Brain Struct. Funct..

[91] Zheng Y., Wang D., Ye Q., Zou F., Li Y., Kwok S.C. (2021). Diffusion property and functional connectivity of superior longitudinal fasciculus underpin human metacognition. Neuropsychologia.

[92] Robertson E.M., Théoret H., Pascual-Leone A. (2003). Studies in cognition: the problems solved and created by transcranial magnetic stimulation. J. Cogn. Neurosci..

[93] Moscovitch M., Cabeza R., Winocur G., Nadel L. (2016). Episodic Memory and Beyond: The Hippocampus and Neocortex in Transformation. Annu. Rev. Psychol..

[94] Vincent J.L., Snyder A.Z., Fox M.D., Shannon B.J., Andrews J.R., Raichle M.E., Buckner R.L. (2006). Coherent spontaneous activity identifies a hippocampal-parietal memory network. J. Neurophysiol..

[95] Chen H.Y., Gilmore A.W., Nelson S.M., McDermott K.B. (2017). Are There Multiple Kinds of Episodic Memory? An fMRI Investigation Comparing Autobiographical and Recognition Memory Tasks. J. Neurosci..

[96] Rotem-Turchinski N., Ramaty A., Mendelsohn A. (2019). The opportunity to choose enhances long-term episodic memory. Memory.

[97] Teghil A., Bonavita A., Procida F., Giove F., Boccia M. (2022). Temporal Organization of Episodic and Experience-near Semantic Autobiographical Memories: Neural Correlates and Context-dependent Connectivity. J. Cogn. Neurosci..

[98] Monsa R., Peer M., Arzy S. (2020). Processing of Different Temporal Scales in the Human Brain. J. Cogn. Neurosci..

[99] Boccia M., Teghil A., Guariglia C. (2019). Looking into recent and remote past: Meta-analytic evidence for cortical re-organization of episodic autobiographical memories. Neurosci. Biobehav. Rev..

[100] Daviddi S., Pedale T., St Jacques P.L., Schacter D.L., Santangelo V. (2023). Common and distinct correlates of construction and elaboration of episodic-autobiographical memory: An ALE meta-analysis. Cortex.

[101] Rossi S., Hallett M., Rossini P.M., Pascual-Leone A., Safety of T.M.S.C.G. (2009). Safety, ethical considerations, and application guidelines for the use of transcranial magnetic stimulation in clinical practice and research. Clin. Neurophysiol..

[102] Kwok S.C., Macaluso E. (2015). Scale invariance of temporal order discrimination using complex, naturalistic events. Cognition.

[103] Gallistel C.R., Gibbon J. (2000). Time, rate, and conditioning. Psychol. Rev..

[104] Montchal M.E., Reagh Z.M., Yassa M.A. (2019). Precise temporal memories are supported by the lateral entorhinal cortex in humans. Nat. Neurosci..

[105] Iyer M.B., Schleper N., Wassermann E.M. (2003). Priming stimulation enhances the depressant effect of low-frequency repetitive transcranial magnetic stimulation. J. Neurosci..

[106] Thut G., Pascual-Leone A. (2010). A review of combined TMS-EEG studies to characterize lasting effects of repetitive TMS and assess their usefulness in cognitive and clinical neuroscience. Brain Topogr..

[107] Kwok S.C., Shallice T., Macaluso E. (2012). Functional anatomy of temporal organisation and domain-specificity of episodic memory retrieval. Neuropsychologia.

[108] Bonnì S., Veniero D., Mastropasqua C., Ponzo V., Caltagirone C., Bozzali M., Koch G. (2015). TMS evidence for a selective role of the precuneus in source memory retrieval. Behav. Brain Res..

[109] Kraft A., Dyrholm M., Kehrer S., Kaufmann C., Bruening J., Kathmann N., Bundesen C., Irlbacher K., Brandt S.A. (2015). TMS over the right precuneus reduces the bilateral field advantage in visual short term memory capacity. Brain Stimul..

[110] Mancini M., Mastropasqua C., Bonnì S., Ponzo V., Cercignani M., Conforto S., Koch G., Bozzali M. (2017). Theta Burst Stimulation of the Precuneus Modulates Resting State Connectivity in the Left Temporal Pole. Brain Topogr..

[111] Friston K.J., Penny W., Phillips C., Kiebel S., Hinton G., Ashburner J. (2002). Classical and Bayesian inference in neuroimaging: theory. Neuroimage.

[112] Penny W., Holmes A., Function H.B., Frackowiak S.J.R., Friston J.K., Frith D.C., Dolan J.R., Price J.C., Zeki S., Ashburner T.J., Penny D.W. (2004). Random-Effects Analysis.

[113] Talairach J., Tournoux P. (1988).

[114] Tzourio-Mazoyer N., Landeau B., Papathanassiou D., Crivello F., Etard O., Delcroix N., Mazoyer B., Joliot M. (2002). Automated anatomical labeling of activations in SPM using a macroscopic anatomical parcellation of the MNI MRI single-subject brain. Neuroimage.

[115] Singmann H., Bolker B., Westfall J., Aust F. (2015). afex: Analysis of factorial experiments. R package version 0.

