# Supplementary material for: Context-dependent abstraction of temporal relationships during naturalistic episodic memory
Source: iScience. 2026 Sep 4;29(9):117391. doi: 10.1016/j.isci.2026.117391 (PMC13571734; doi:10.1016/j.isci.2026.117391)
Supplement: Supplementary file 1 [file mmc1.pdf]

iScience, Volume *29*

Supplemental information

Context-dependent abstraction of temporal relationships during naturalistic episodic memory

Qun Ye, Yi Hu, Yixuan Ku, Kofi Appiah, and Sze Chai Kwok

Supplementary information

This Supplemental material contains seven (7) Supplementary Figures, one (1) Supplementary Table, and two (2) Supplementary Text.

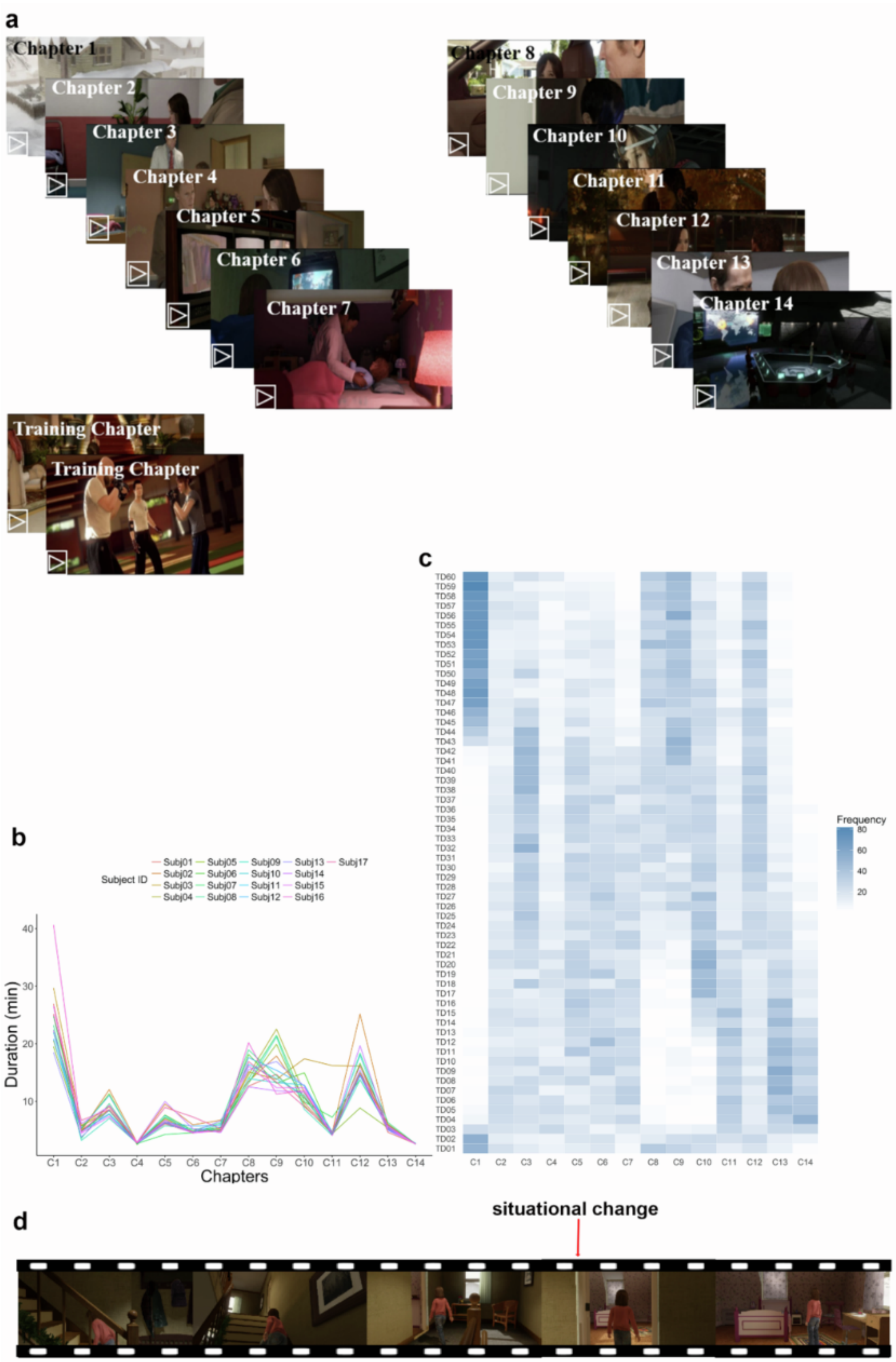

Figure S1 Stimuli for encoding and related analyses. (a) 14 different

chapters were used: seven in each of two within-subjects TMS sessions (see also **Table S1**). **(b)** Playing durations varied across chapters and subjects. **(c)** Intensity map showed the numbers of trials extracted from the 14 game chapters separately for the 60 TD levels across subjects. The numbers of image pairs extracted for the TOJ task were approximately equal across the chapters, except that longer TD are more likely to be extracted from the longest chapter (e.g., C1). However, in a control analysis, we removed trials from the longest 1/6 of the TD distribution to minimize effects due to long TDs on behavioral results (see subsection on **Control analysis on removing effects from long TDs**). **(d)** An excerpt of a subject-specific video illustrates how situational changes were defined frame-by-frame for Situational Change RDMs and pmod analyses. Noting that time and space in our task were partially correlated, we quantified subject-specific situational changes in terms of the number of locations each participant had traversed in the video (i.e., spatial displacement embedded in the paired images presented in TOJ task).

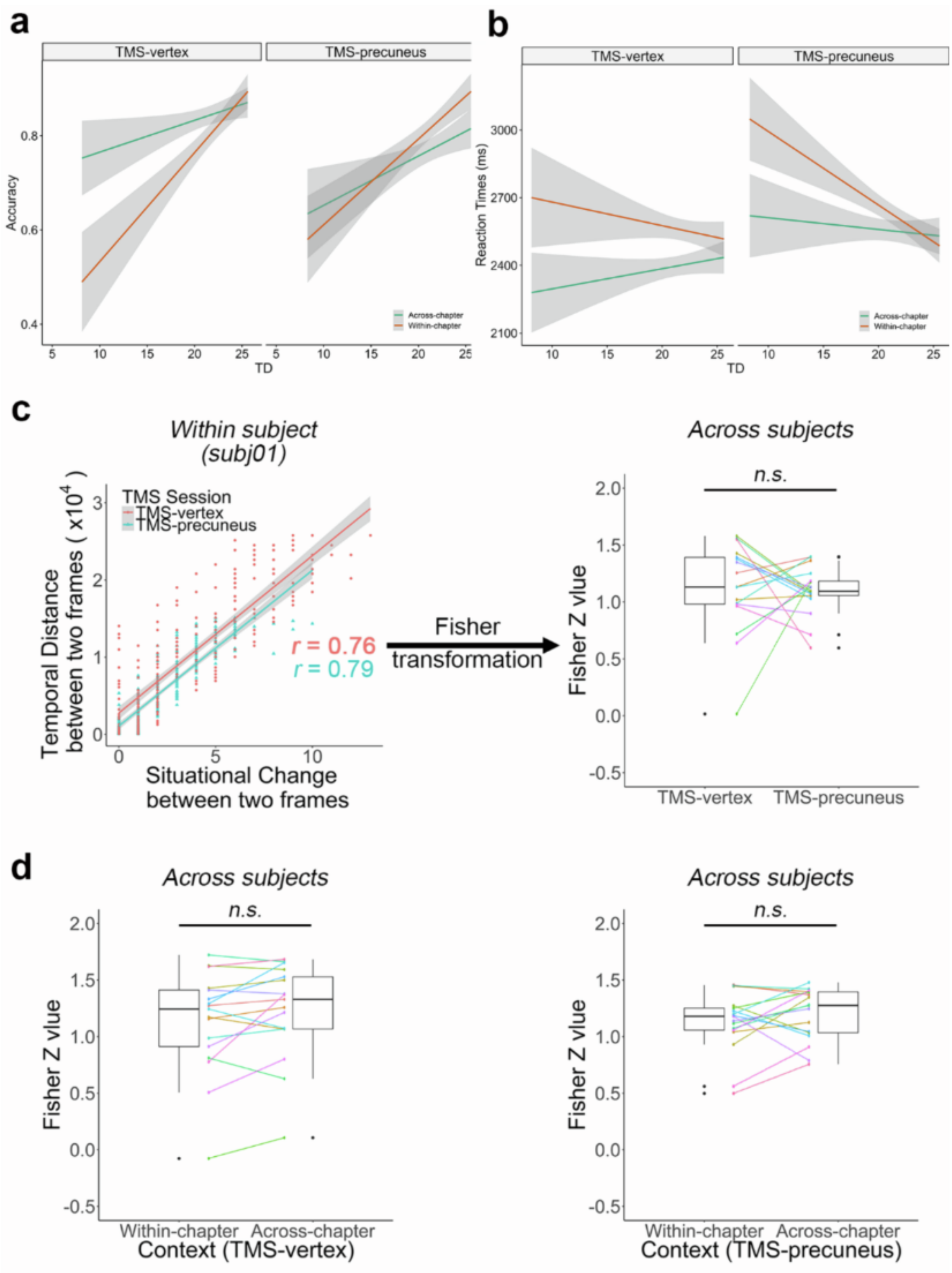

Figure S2 Behavioral results. (a) Estimated probability of accurate temporal order judgments (TOJ) as a function of temporal distance (TD centered), Context (Across-chapter vs. Within-chapter), and TMS session (vertex vs.

precuneus) with only high confidence trials included. TMS session significantly influenced accuracy, $\chi^2_{(1)}$ = 4.17, *P* = .041, and a significant TD × Context interaction was observed, $\chi^2_{(1)}$ = 7.18, *P* = .007. Follow-up analyses indicated a steeper effect of TD in the Within-chapter condition (slope = 0.65, 95% CI [0.47, 0.83]) compared to the Across-chapter condition (slope = 0.32, 95% CI [0.16, 0.49], *P* = .007). Furthermore, TMS effects varied across contexts, as vertex TMS led to higher accuracy in the Across-chapter context (*P* = .026), whereas precuneus TMS resulted in lower accuracy in the Across-chapter context (*P* = .002). **(b)** Estimated reaction times (in milliseconds) as a function of temporal distance (TD centered), Context (Across-chapter vs. Within-chapter), and TMS session (vertex vs. precuneus) with only high confidence trials included.Significant main effects of TD (*P* = .034), Context (*P* < .001), and TMS session (*P* < .001) were observed. Interactions included TD × Context (*P* < .001), TD × TMS session (*P* = .049), and Context × TMS session (*P* = .006), but no three-way interaction was found (*P* = .819). Follow-up analyses showed that TD effects were positive for Across-chapter (slope = 0.03, 95% CI [-0.02, 0.08]) and negative for Within-chapter (slope = -0.11, 95% CI [-0.17, -0.06]; *P* < .001). TD effects were more pronounced for precuneus stimulation (slope = -0.08, 95% CI [-0.13, -0.03]) compared to vertex stimulation (slope = 0, 95% CI [-0.06, 0.05]; *P* = .005). Differences in reaction times between contexts were significant for vertex TMS (*P* < .001) but not precuneus TMS (*P* = .11). **(c)** High correlation coefficients between temporal distance and number of situational

changes across trials in all subjects. Correlation coefficients are highly correlated for all participants in both TMS sessions (each dot represents a trial; one participant illustrated as an example, left panel). Fisher *z* values (transformed from correlation coefficients) for all participants are shown in a boxplot on the right side. Fisher *z* values of two conditions are significantly greater than zero (TMS-vertex session: mean ± se = 1.12 ± 0.1, $t_{16} = 11.52$, $P = 3.71 \times 10^{-9}$; TMS-precuneus session: mean ± se = 1.10 ± 0.05, $t_{16} = 21.08$, $P = 4.24 \times 10^{-13}$). No significant differences exist between the two sessions ($P = 0.83$). Individual participants' values are connected between the two boxplots. **(d)** For completeness, we also calculated the Fisher *z* values for all participants in the Across-chapter and Within-chapter conditions of two TMS sessions separately. *Z* values are significantly greater than zero in each condition ($P < 0.01$). No significant differences exist between the two sessions ($P > 0.05$). *n.s.* denotes not significant.

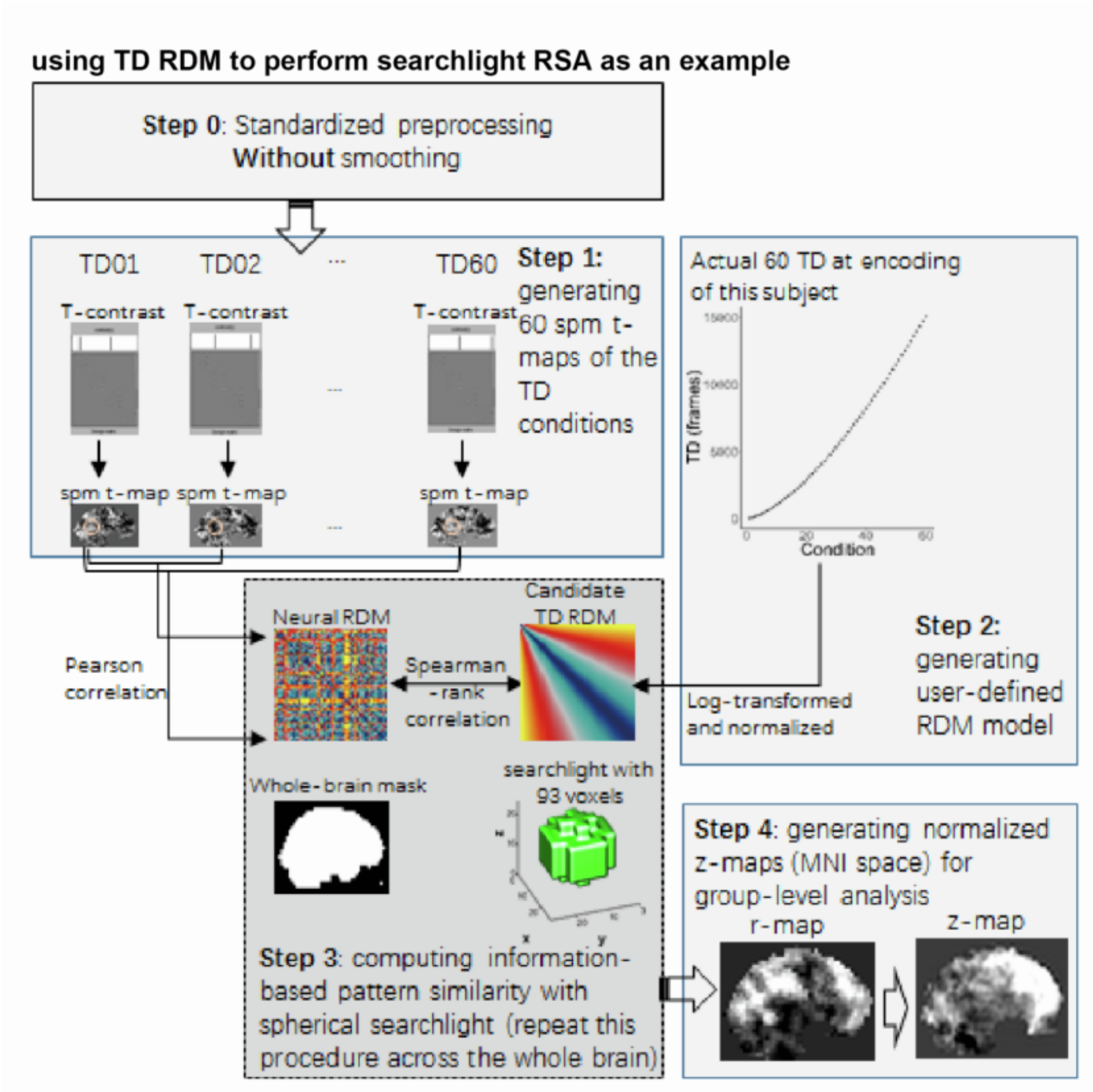

Figure S3 Pipeline of RSA analysis. Using one model (TD RDM) to perform the searchlight procedure as an example in one subject (Subj01). The same principle and procedure applied to all other candidate RDMs and subjects.

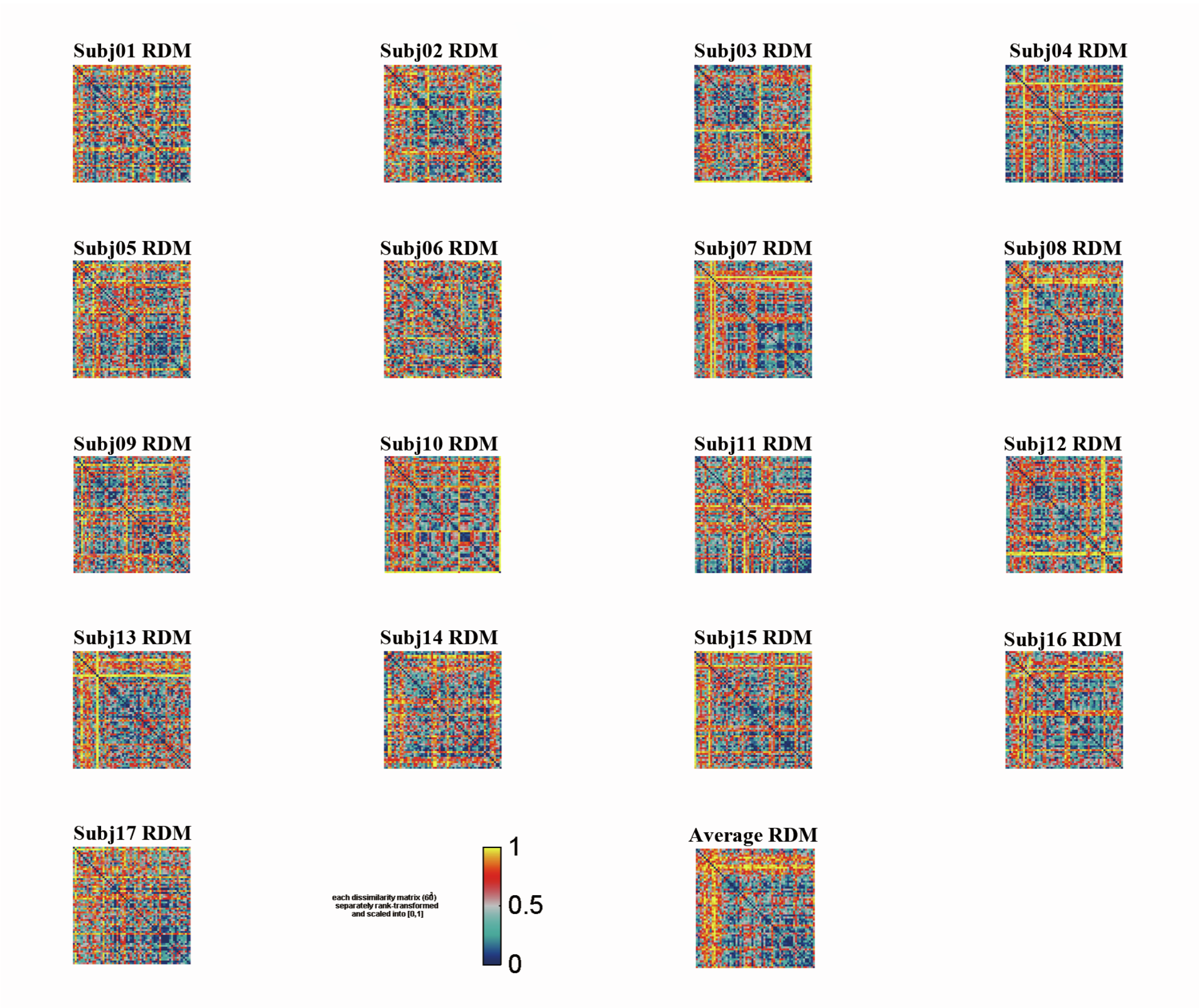

Figure S4. Individuals and their group-averaged neural RDMs. Only Within-chapter conditions are displayed.

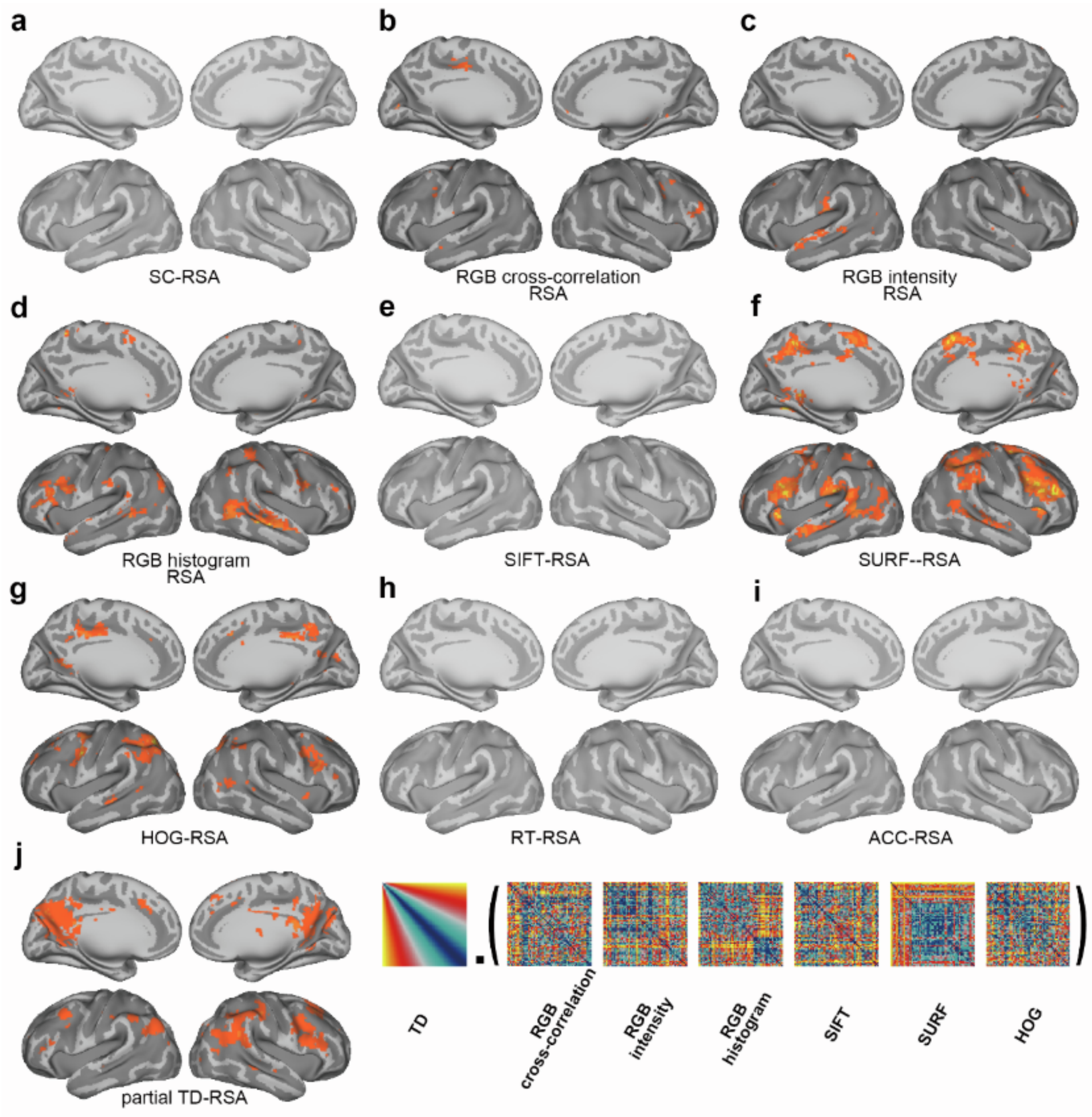

Figure S5 RSA searchlight results for TMS-vertex sessions using candidate RDMs. (a) Situational Change (SC) RDM (cf. **Fig. S1d**). **(b)** RGB cross-correlation RDM. **(c)** RGB-intensity RDM. **(d)** RGB-histogram RDM. **(e)** SIFT RDM. **(f)** SURF RDM. **(g)** HOG RDM. **(h)** Reaction time RDM. **(i)** Accuracy RDM. **(j)** A partial correlation analysis showed that the TD representation in the posteromedial parietal areas was not subject to influences due to the six perceptual RDMs. MRI results are displayed at $P_{uncorrected} < 0.001$.

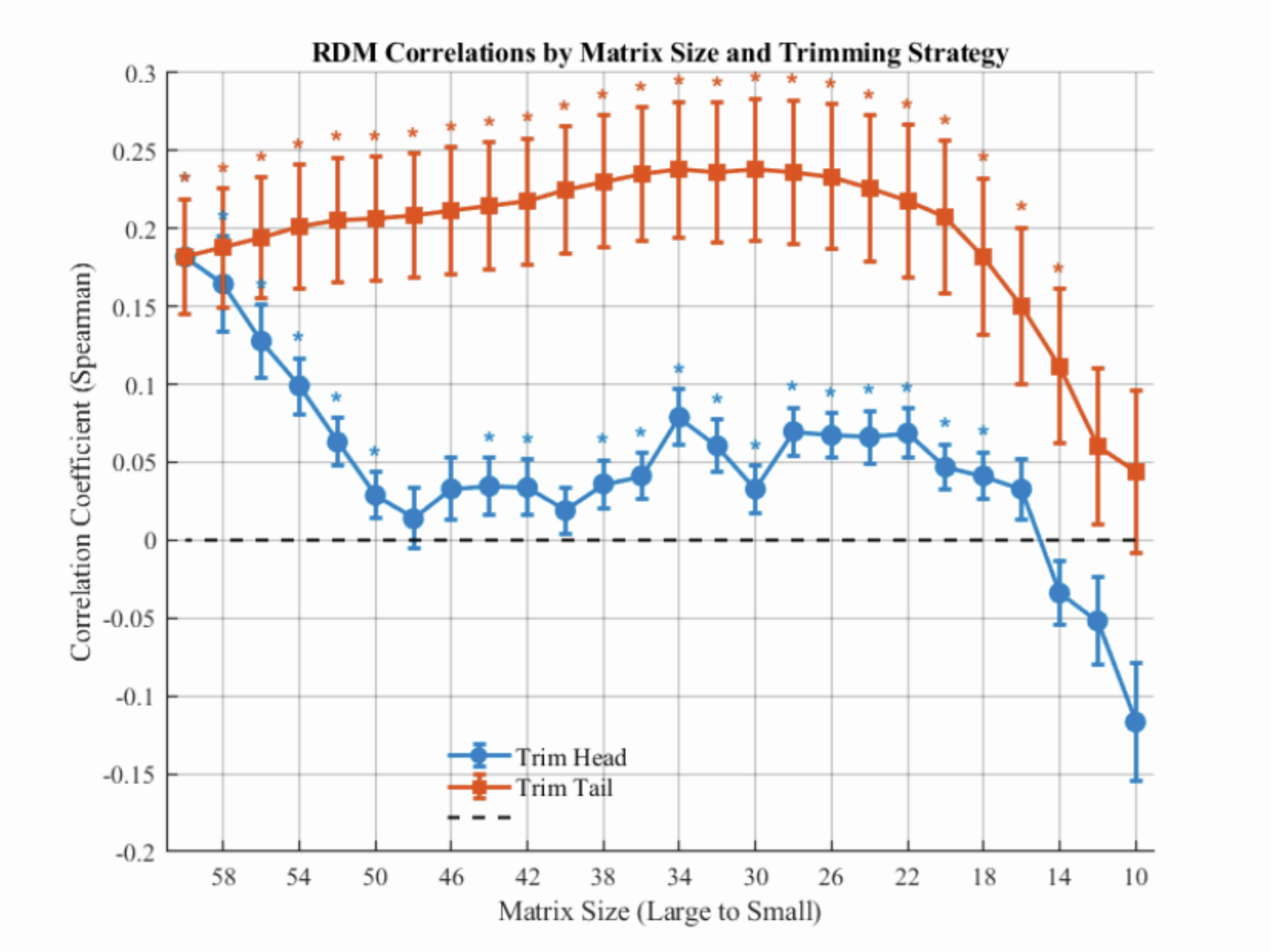

Figure S6 Robustness of temporal distance (TD) representational similarity analysis (RSA) across matrix trimming strategies. Spearman correlation coefficients between theoretical TD representational dissimilarity matrices (RDMs) and observed neural RDMs extracted from the precuneus region of interest, plotted as a function of matrix size. Orange line (Trim Tail): correlations computed after sequentially removing the longest TDs, starting from the full 60 × 60 matrix and progressing to smaller matrices by excluding longer temporal distances. Blue line (Trim Head): correlations computed after sequentially removing the shortest TDs, starting from the full matrix and progressing by excluding shorter temporal distances. For each iteration, we computed Spearman correlations between the model Log TD RDM (log-transformed differences) and the empirical neural RDM (reference RDM). We

present the Spearman correlation coefficients as a function of decreasing matrix size for each strategy. For the Trim Tail strategy (orange line), correlations started low for the full matrix and increased initially as long TDs were removed, peaking around matrix sizes of 34 ($r = 0.24 \pm 0.04$, $p < .05$ FDR-corrected), before declining for very small matrices (e.g., size 12: $r = .06$, n.s.). This suggests that TD coding remains robust when excluding the longest TDs, which is potentially confounded by long Chapters over-representation. In contrast, for the Trim Head strategy (blue line), correlations started high for the full matrix ($r = .18 \pm 0.04$, $p < .05$ FDR-corrected) and remained positive and significant, before dropping sharply and becoming negative for the smallest matrices (e.g., size 10: $r = -.12$, n.s.). Overall, these results demonstrate that the multivoxel TD coding in the precuneus is not solely driven by extremely short- or long-TD pairs, or by long-vs-short comparisons. Significant correlations persist across a range of matrix sizes, even after trimming the tail (long TDs), supporting the generalizability of our findings beyond potential chapter-specific confounds. Error bars represent standard error of the mean across participants. Asterisks (*) indicate correlations significantly greater than zero ($p < .05$, FDR-corrected). See also **Supplementary Text S1**.

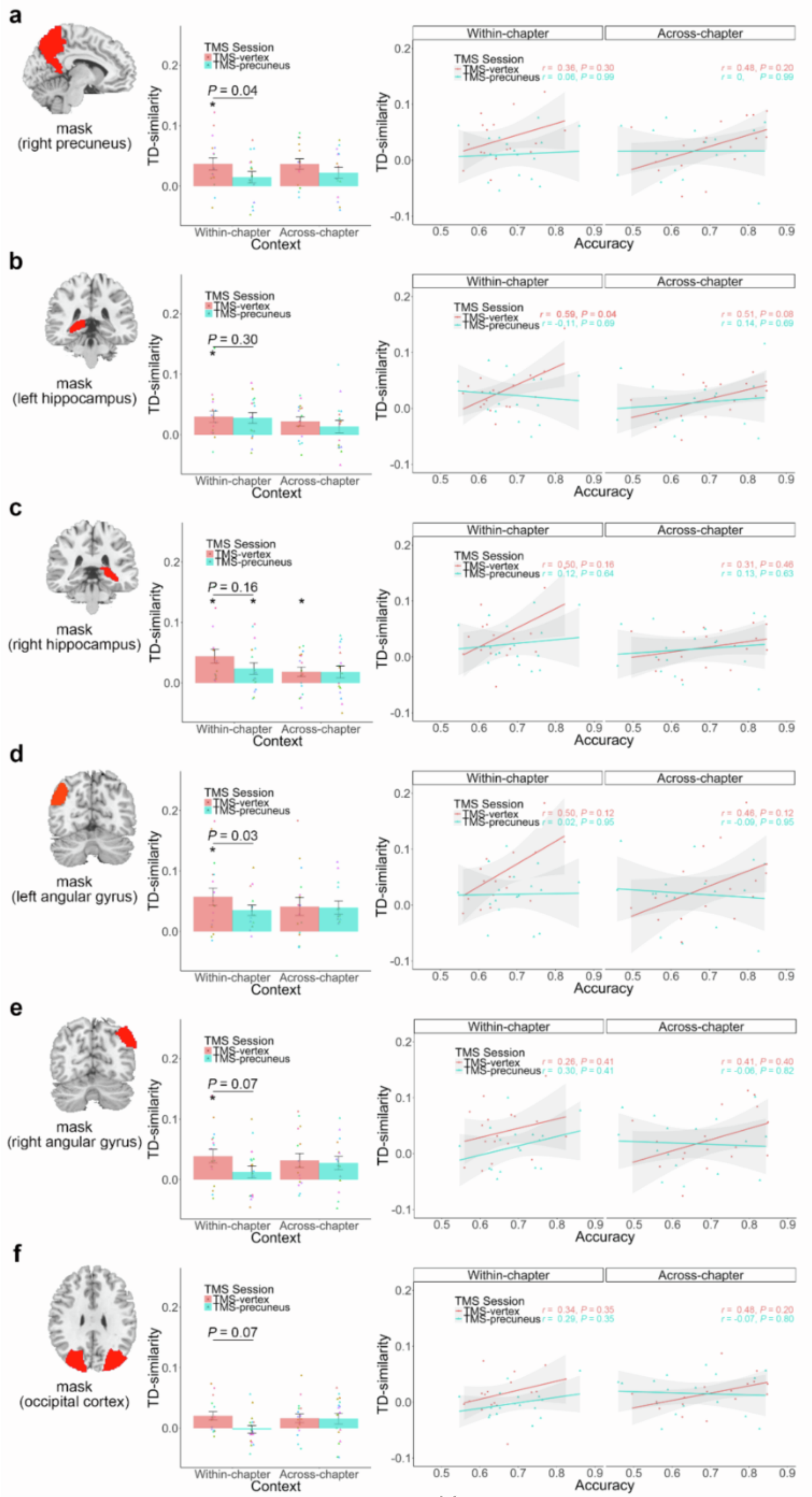
a
mask
(right precuneus)
TMS Session
TMS-vertex
TMS-precuneus
P = 0.04
TD-similarity
Within-chapter
Across-chapter
Context
r = 0.36, P = 0.30
r = 0.06, P = 0.99
r = 0.48, P = 0.20
r = 0, P = 0.99
Accuracy
b
mask
(left hippocampus)
P = 0.30
r = 0.59, P = 0.04
r = -0.11, P = 0.69
r = 0.51, P = 0.08
r = 0.14, P = 0.69
c
mask
(right hippocampus)
P = 0.16
r = 0.50, P = 0.16
r = 0.12, P = 0.64
r = 0.31, P = 0.46
r = 0.13, P = 0.63
d
mask
(left angular gyrus)
P = 0.03
r = 0.50, P = 0.12
r = 0.02, P = 0.95
r = 0.46, P = 0.12
r = -0.09, P = 0.95
e
mask
(right angular gyrus)
P = 0.07
r = 0.26, P = 0.41
r = 0.30, P = 0.41
r = 0.41, P = 0.40
r = -0.06, P = 0.82
f
mask
(occipital cortex)
P = 0.07
r = 0.34, P = 0.35
r = 0.29, P = 0.35
r = 0.48, P = 0.20
r = -0.07, P = 0.80

Figure S7 TD-similarity indices in six anatomical ROIs. (**a**: right precuneus, **b-c**: bilateral hippocampi, **d-e**: bilateral angular gyrus, and **f**: whole occipital cortex) separately for Within-chapter and Across-chapter conditions, in both TMS sessions, and their correlation with individual subjects TOJ accuracy. * denotes $P < 0.05$, FDR-corrected, one-sample t-test against zero. Horizontal lines indicate paired t-test between two bars and P values are FDR-corrected.

Table S1. Descriptive statistics of TMS motor threshold and full counterbalancing for gameplay chapters by TMS sites.

ID	Motor threshold	Session 1			Delay (days)	Session 2		
		Chapters (Playing)	Duration (min)	TMS Site		Chapters (Playing)	Duration (min)	TMS Site
Subj01	66%	Chap 1 ~ 7	89.39	Precuneus	9	Chap 8 ~ 14	35.19	Vertex
Subj02	67%	Chap 1 ~ 7	102.70	Vertex	6	Chap 8 ~ 14	44.46	Precuneus
Subj03	80%	Chap 8 ~ 14	35.53	Vertex	9	Chap 1 ~ 7	114.21	Precuneus
Subj04	58%	Chap 8 ~ 14	36.41	Vertex	2	Chap 1 ~ 7	94.70	Precuneus
Subj05	65%	Chap 1 ~ 7	80.50	Precuneus	8	Chap 8 ~ 14	35.31	Vertex
Subj06	67%	Chap 1 ~ 7	95.05	Vertex	3	Chap 8 ~ 14	39.63	Precuneus
Subj07	72%	Chap 8 ~ 14	32.41	Vertex	4	Chap 1 ~ 7	97.06	Precuneus
Subj08	73%	Chap 1 ~ 7	96.08	Precuneus	8	Chap 8 ~ 14	37.03	Vertex
Subj09	69%	Chap 1 ~ 7	88.81	Vertex	7	Chap 8 ~ 14	31.46	Precuneus
Subj10	80%	Chap 8 ~ 14	35.69	Precuneus	7	Chap 1 ~ 7	89.79	Vertex
Subj11	65%	Chap 8 ~ 14	38.77	Vertex	26	Chap 1 ~ 7	86.02	Precuneus
Subj12	71%	Chap 1 ~ 7	90.56	Precuneus	16	Chap 8 ~ 14	35.89	Vertex
Subj13	68%	Chap 8 ~ 14	35.66	Vertex	7	Chap 1 ~ 7	87.98	Precuneus
Subj14	73%	Chap 1 ~ 7	87.94	Precuneus	7	Chap 8 ~ 14	40.56	Vertex
Subj15	57%	Chap 8 ~ 14	32.72	Precuneus	5	Chap 1 ~ 7	91.46	Vertex
Subj16	69%	Chap 8 ~ 14	35.44	Vertex	5	Chap 1 ~ 7	108.49	Precuneus
Subj17	61%	Chap 1 ~ 7	91.10	Precuneus	6	Chap 8 ~ 14	42.13	Vertex

Supplementary Text S1. Differential contributions by short and long TDs to the memory representational structure. Related to Figure S6.

It is interesting to note that on a log-transformed temporal scale, short and long TDs contribute very differently to the representational structure. Short TDs (e.g., TD1–10) are spread across a wide range in log space—for example, the log difference between TD1 and TD10 is substantial—so these intervals are naturally more distinguishable from one another and from all other TDs. In contrast, long TDs are compressed on a log scale (e.g., TD50 and TD60 differ very little), making them inherently more similar to each other. Moreover, the longest TDs disproportionately originate from long Chapters, which raises the possibility that the step-like pattern at the upper end might reflect chapter structure. Our Trim Tail analysis addresses this directly: removing the longest TDs (TD51–60), including those tied to long Chapters, does not eliminate the TD representation, indicating that the pattern at the long end is not driving the core effect. Conversely, removing the shortest TDs (TD1–10) in the Trim Head analysis markedly reduces the correlations. This confirms that short TDs—where the representational gradient is steepest—anchor the temporal structure, consistent with the known compressive/logarithmic coding of interval magnitude.

The "step-like" shape we see at the two ends of the temporal-distance curve reflects two different things: First, at the long-TD end, the pattern is partly an artifact — it arises because long intervals have very little discriminable variance

in a logarithmically compressed representation. The model (and the brain) simply cannot distinguish those bins well, so they all look similarly low. This makes that end appear “flat” or “stepped,” but it does not carry strong functional information. Second, at the short-TD end, the step-like pattern is foundational — this is where the representational gradient is strongest. Short distances contain the highest-resolution temporal information, so any apparent jumps or strong slopes here are real and reflect the core temporal-order structure the brain encodes. In brief, the long end shows a step pattern because of limited resolution (an artifact), whereas the short end shows a step pattern because that region contains the true representational signal (foundational).

Supplementary Text S2. Description of Model 3 to Model 10: Candidate perceptual models (RGB cross-correlation, RGB intensity, RGB histogram, SIFT, SURF, HOG) and behavioral models (RT, ACC)

Model 3 (RGB-cross-correlation RDM, 60 × 60), *Model 4* (RGB-intensity RDM, 60 × 60) and *Model 5* (RGB-histogram RDM, 60 × 60) considered the perceptual characteristics of the images used in TOJ. For Model 3, the similarity measure was based on the cross-correlation value between two images (image of size 1920 × 1080) for the three color channels (red, green, and blue; RGB). For every pair of images in each of the three color channels (RGB), we computed the cross-correlation coefficients between the pair. This is a measure of the displacement of one image relative to the other; the larger the cross-correlation coefficient (which ranges between -1 and 1), the more similar the two images were. We then computed the differences with and among every other condition producing 60 × 59/2 values, which were then assigned to the corresponding cells of the RDM. For Model 4, we computed the pixel-wise difference between pair images for the three color channels (RGB). The computed difference is useful when the compared images are taken from a stationary camera with infinitesimal time difference. The output pixel for each color channel is assigned with the value 1 if the absolute difference between the corresponding pixels in the image pair is non-zero, or a value of 0 otherwise. A single value is generated for each of the three color channels by summing all the output pixel values (either 0 or 1). We averaged the sum of difference for

all three color-channels for the intensity value of each pair of images and then computed the differences with and among every other condition producing 60 × 59/2 values, which were then assigned to the corresponding cells of the RDM. For Model 5, we constructed color histograms for image pairs and computed the Sum-of-Square-Difference (SSD) error between them for the three color channels (RGB). For each color channel the intensity values range from 0 to 255 (i.e., 256 bins). We first computed the total number of pixels at each intensity value and then computed the SSD for all 256 bins for each image pair. The smaller the value of the SSD, the more similar the two images (image pair) were. We then computed the differences with and among every other condition producing 60 × 59/2 values, which were then assigned to the corresponding cells of the RDM. In contrast to model 4, this approach does not require corresponding pixels in the image pair to be the same but rather measures the existence of pixel intensity in both images. Overall, the three perceptual-similarity models (3, 4 and 5) look at different similarity measures, and they complement each other; thus, any difference in the appearance of the two images irrespective of the temporal distance, could be accounted for by at least one of the three models. For any two similar images, the RGB-intensity RDM results in a very small value, thus the corresponding pixels are virtually the same for the entire image. The RGB-histogram will also result in a small value as the image pairs will have the same histogram bins. The RGB-cross-correlation value will be close to 1, signifying the similarity in the images. When

subsections of a scene are visible in both images with varied brightness, the RGB-cross-correlation value will still be closer to 1 but with a very high RGB-intensity RDM value.

Model 6 (Scale Invariant Feature Transform (SIFT) RDM, 60 × 60), *Model 7* (Speeded Up Robust Features (SURF) RDM, 60 × 60) and *Model 8* (Histogram of Oriented Gradients (HOG), 60 × 60): For model 6, SIFT transform images into scale-invariant vectors which encoded interest points. For any pair of images to be compared, a computational search over selected scales and image locations is conducted using Difference-of-Gaussian (DoF) to identify potential interest points that are invariant to scale and orientation in each image. Interest points from each image are stabilized using Taylor series expansion of scale space to get a more accurate location of extrema. This is followed by the construction of an orientation histogram to achieve invariance to rotation. Key-points between the two images are then matched by identifying their nearest neighbors using minimum Euclidean distance between the invariant descriptor vectors extracted from each image. For model 7, rather than the use of DoF in SIFT[53] as an approximation of Laplacian-of-Gaussian (LoG), SURF[54] uses Box Filter which is calculated in parallel using integral images[55] to approximate LoG. Wavelet responses in both horizontal and vertical directions are used to assign orientation in SURF. Like SIFT, the first step in SURF consists of fixing a reproducible orientation based on information from a circular region around the interest point. A descriptor vector is generated around the interest point using

an integral image, which is compared to descriptor vectors extracted from a compared image to find a match. The Euclidean distance has been used to measure the similarity between two descriptor vectors from the two images. It is worth noting that SURF was not used in either Greene et al.[56] or Aminoff et al[57] but it is robust and much faster to compute than SIFT. However, Greene et al.[56] trained a state-of-the-art convolutional neural network (CNN) on ImageNet 2012 to find visual features to perform their categorization. To remove the time for training a supervised network like CNN which uses SIFT/SURF features in its layers, our dissimilarity measures are based on the features extracted solely from the input images with no prior training. For model 8, in the case of the HOG feature descriptor, we constructed a histogram of directions of gradient over fixed sized grid across the entire image. A vector is generated from each grid cell and correlated with HOG features from another image. It is worth noting that gradients or derivatives of x and y (location) in an image are useful for interest point localization because they have higher values around edges and corners, which hold more information about objects.

Model 9 (Reaction time, or RT RDM, 60 × 60) and *Model 10* (Accuracy, or ACC RDM, 60 × 60) were computed based on the behavioral performance of reaction times and percentage correct (0 or 1) for each trial. We averaged the values along the 60 TD levels first (4 repetitions when Within-chapter and Across-chapter conditions collapsed; 2 repetitions for Within-chapter or Across-chapter alone) and computed the differences with and among every other TD

levels producing 60 × 59/2 values, which were then assigned to the corresponding cells of the RT RDM and ACC RDM.
